# A Systems Vaccinology Approach Reveals the Mechanisms of Immunogenic Responses to Hantavax Vaccination in Humans

**DOI:** 10.1038/s41598-019-41205-1

**Published:** 2019-03-18

**Authors:** Adnan Khan, Ok Sarah Shin, Jinhyuk Na, Jae Kwan Kim, Rak-Kyun Seong, Man-Seong Park, Ji Yun Noh, Joon Young Song, Hee Jin Cheong, Youngja Hwang Park, Woo Joo Kim

**Affiliations:** 1 Metabolomics Laboratory, Korea University College of Pharmacy, Sejeong city, Republic of Korea; 2 Department of Biomedical Sciences, Korea University College of Medicine, Seoul, Republic of Korea; 3 Department of Microbiology, Korea University College of Medicine, Seoul, Republic of Korea; 4 Division of Infectious Diseases, Department of Internal Medicine, Korea University College of Medicine, Seoul, Republic of Korea

## Abstract

Hantavax is an inactivated vaccine for hemorrhagic fever with renal syndrome (HFRS). The immunogenic responses have not been elucidated yet. Here we conducted a cohort study in which 20 healthy subjects were administered four doses of Hantavax during 13-months period. Pre- and post- vaccinated peripheral blood mononuclear cells (PBMCs) and sera were analysed by transcriptomic and metabolomic profilings, respectively. Based on neutralizing antibody titers, subjects were subsequently classified into three groups; non responders (NRs), low responders (LRs) and high responders (HRs). Post vaccination differentially expressed genes (DEGs) associated with innate immunity and cytokine pathways were highly upregulated. DEG analysis revealed a significant induction of CD69 expression in the HRs. High resolution metabolomics (HRM) analysis showed that correlated to the antibody response, cholesteryl nitrolinoleate, octanoyl-carnitine, tyrosine, ubiquinone-9, and benzoate were significantly elevated in HRs, while chenodeoxycholic acid and methyl palmitate were upregulated in NRs and LRs, compared with HRs. Additionally, gene-metabolite interaction revealed upregulated gene-metabolite couplings in, folate biosynthesis, nicotinate and nicotinamide, arachidonic acid, thiamine and pyrimidine metabolism in a dose dependent manner in HR group. Collectively, our data provide new insight into the underlying mechanisms of the Hantavax-mediated immunogenicity in humans.

## Introduction

Systems vaccinology approaches have enabled us to better understand vaccine-induced protective immune responses in humans^1–7^. Very recently, high-throughput RNA sequencing (RNA-seq) technology, a powerful tool for profiling the transcriptome, has been employed in various viral infections and diseases^8^. Furthermore, the systemic analysis of metabolomic data can link known metabolites with genes via their shared metabolic reactions and pathways, thereby enhancing the integration and significance of transcriptomic and metabolomic data^9^. Integrative transcriptomic and metabolomic analyses have been applied as systems vaccinology tools. This approach has the potential to reveal the alteration dynamics of host gene and metabolite expression in the immune response to vaccination, which could help uncover predictive markers for vaccine immunogenicity and effectiveness.

Haemorrhagic fever with renal syndrome (HFRS) caused by hantaviruses is widely distributed throughout Asia and Europe^10,11^. HFRS has distinct clinical and pathological features, including high fever, thrombocytopenia, increased capillary permeability, and up-regulation of tumour necrosis factor-alpha (TNF-α)^12^, however, the mechanisms of hantavirus-induced pathogenesis are not fully understood. Hantaviruses establish chronic infections in rodents and are transmitted to humans via contact with feces, urine, or saliva of infected mice^10^. Therefore, the risk factors for HFRS increased among people of professions such as forestry and farming as well as in military personnel^13^.

Hantavax is a commercialized inactivated vaccine used for preventing HFRS in South Korea since 1990^14^. We previously evaluated long-term immunogenicity and safety of Hantavax in a phase III, multi-center clinical trial, with a three-dose vaccination at 0-1- and 13-month timepoints among healthy adults^14^. Anti-Hantaan virus (HTNV) neutralizing antibody titers were found to be low after the first two doses, but increased after a booster dose, suggesting that vaccinations for three times might be required for enhancing the neutralizing antibody titers.

In the present study, a four-dose schedule was employed with primary vaccination for three times, followed by one booster vaccination. We used transcriptomic and metabolomic analysis to comprehensively assess vaccine-induced protective immune responses. We performed bioinformatics analyses to delineate the kinetics of vaccine-induced immunity, to identify the dynamics of enriched modules over time, and to determine whether and how transcriptomic and metabolomic data correlate with neutralizing antibody responses.

## Results

### Study design for integrative profiling of Hantavax immunogenicity in humans

A phase III, multi-center, open labelled immunogenicity study of Hantavax vaccination was conducted in Guro Hospital of Korea University College of Medicine between November 2015 and January 2017. Twenty healthy adults were initially enrolled, of which one was excluded after showing a high neutralizing titer in pre-vaccination serum. Thus 19 subjects were used in the analysis of the results. Exclusion criteria for participants were: previous hantavirus infection, previous hantavirus vaccination, positive neutralizing antibody response at screening, allergy to vaccine components, history of seizure within the past year, pregnant or breastfeeding women, acute febrile illness, marked nutritional deficiency, uncontrolled chronic medical conditions (cardiovascular, renal, or hepatic disease), receipt of immunosuppressive or immune modifying drugs, participation in a clinical trial for another vaccine within 30 days prior to enrolment, or other vaccinations within two weeks prior to enrolment in the study.

After a baseline blood samples collection, each participant was administered Hantavax vaccine four times according to the 0-1-2-13 month schedule. The demographic characteristics of the subjects are listed in Table 1. Blood samples were collected before 1^st^ and 72 hours after administration of the 2^nd^, 3^rd^ and 4^th^ doses (Supplementary Fig. S1). Throughout the course of study, we measured HTNV-specific antibody titers using plaque reduction neutralizing antibody test (PRNT50) and immunofluorescent antibody assay (IFA), as described previously^15^. Based on the neutralizing antibody titers following the 4^th^ vaccination (Supplementary Fig. S1), subjects were classified into three groups: NRs, LRs and HRs (Table 1). The distribution was made based on PRNT_50_ titer. The cut-off level for a distinction between HR and LR was set as 1:40, whereas the cut-off level between LR and NR was set as 1:10 for PRNT_50_ titer (HR >1:40, LR 1:10~1:40 and NR <1:10). Systems vaccinology analyses were performed based on two different approaches: vaccination instance and responsiveness to the vaccine.

**Table 1:** Demographic characteristics of vaccinees.

Responder group	Non responders	Low responders	High responders
Vaccinee (*n*)	6	8	5
Gender	F = 3	F = 4	F = 4
	M = 3	M = 4	M = 1
Average age (mean ± SD)	46.4 ± 9.7	42.0 ± 10.6	31.0 ± 9.5

### Analysis of high-throughput sequencing RNA-seq transcriptome

To understand the mechanisms driving Hantavax responses, we performed transcriptomic analyses of PBMCs and high-resolution metabolomics of sera obtained from the vaccinees. More than 37 million 100-bp paired-end reads were generated. The base quality of RNA-seq reads was checked and analyzed using an Agilent RNA 6000 Nano Kit. Each sample had relatively high sequencing coverage range. The uniquely mapped reads, reflecting no introduction of obvious bias, covered over 80% of the total genomic bases (data not shown).

**Figure 1:**
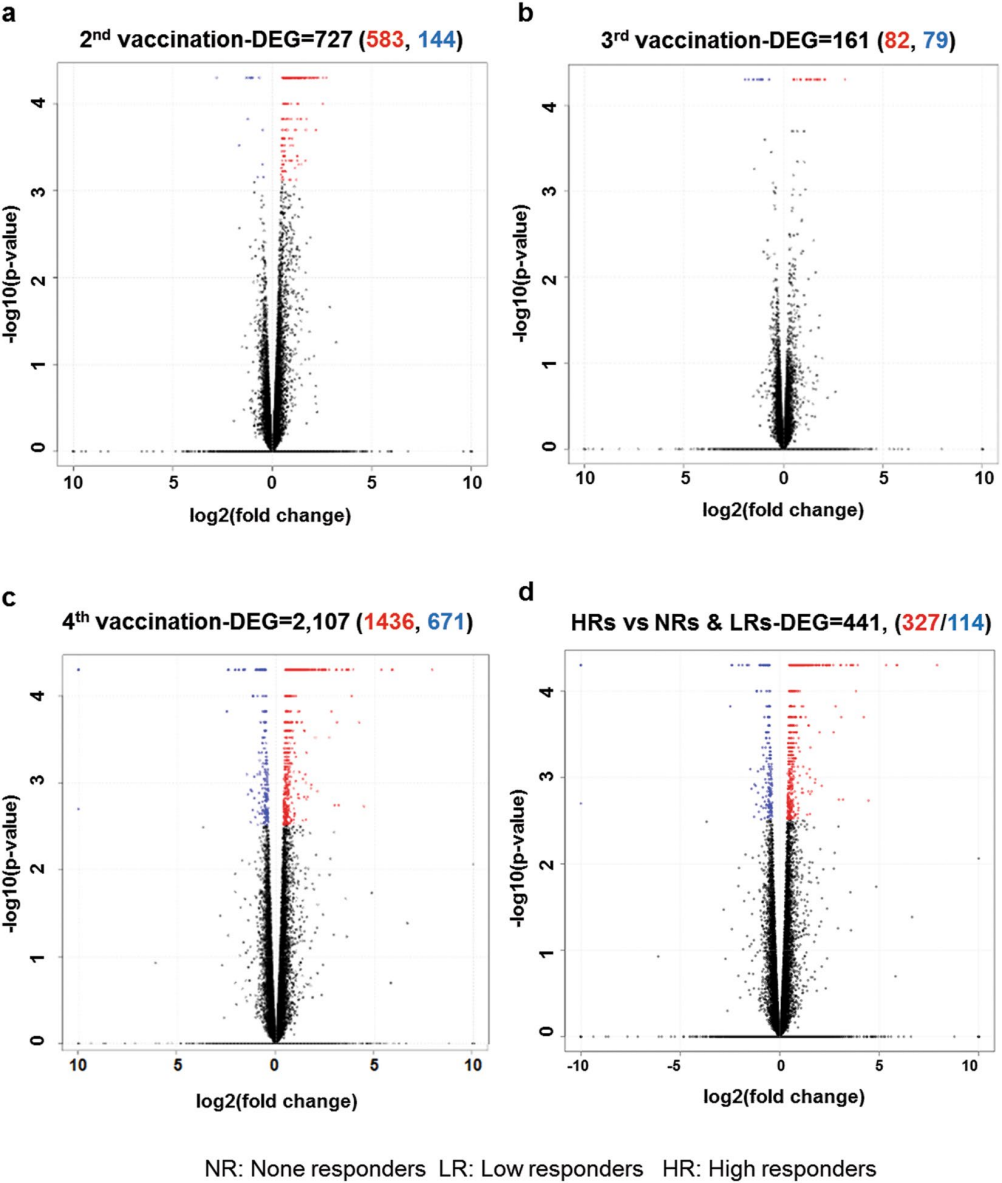
Gene signatures of immunogenicity to Hantavax vaccination based on vaccine responsiveness. Transcriptomic data of the 2^nd^ (**a**), 3^rd^ (**b**), and 4^th^ (**c**) vaccinations relative to pre-vaccination. Numbers of more than two-fold up- or downregulated differentially expressed genes (DEGs) identified from the comparison of the control and virus-infected groups (DEGs were identified based on a false discovery rate (FDR) *q*-value threshold of less than 0.05). The volcano plot shows the DEG patterns for Hantavax vaccinees. (**d**) Vaccinees were classified into three groups based on vaccine responsiveness (i.e. HTNV neutralizing antibody titers): NRs (non-responders), LRs (low responders), and HRs (high responders). The volcano plot shows the DEGs identified based on a false discovery rate (FDR) *q*-value threshold of less than 0.05 for Hantavax vaccinees between HR vs NR & LR. The x-axis represents the log_2_ values of the FC observed for each mRNA transcript, while the y-axis represents the –log_10_ values of the *p*-values of the significance tests between replicates for each transcript. Data for genes that were not classified as defferentially expressed are plotted in black.

Using the 63,193 annotated genes in the human genome database, gene expression was quantified and compared between pre- and post-vaccination groups of each vaccination, and differentially expressed genes (DEGs) with a *q-*value < 0.05 were identified. Supplementary Table S1 shows total numbers of both up- and down-regulated DEGs for LRs vs. HRs, NRs vs. LRs and NRs vs. HRs (with ± log 2-fold changes). The 2^nd^ vaccination sera showed a high number of upregulated genes compared with 3^rd^ or 4^th^ vaccination, which may have been caused due to variation in population among three groups. To assess whether the general expression pattern of transcripts was similarly distributed across vaccinees after each dose, a volcano plot was generated for the PBMC after the 2^nd^ (Fig. 1a), 3^rd^ (Fig. 1b) and 4^th^ vaccine administrations (Fig. 1c). The ratio of differential expression (fold change (FC), illustrated in the abscissa of the volcano plot) showed a very good correlation between the FC differences and *p*-values (i.e. genes with a high FC difference also had a low *p*-value in the group-wise comparison). Interestingly, 4^th^ vaccinated sera (Fig. 1c) were observed with highest number of up-regulated (1,436) and/or down-regulated (671) DEGs, compared to 2^nd^ sera (583 upregulated & 144 downregulate DEGs), and 3^rd^ vaccinated PBMC (82 upregulated & 79 downregulated DEGs). To evaluate the degree of dissimilarity among samples with regard to biological variations and dimensions, we compared transcriptome obtained by RNA-seq after the 1^st^, 2^nd^, 3^rd^ and 4^th^ vaccinations by performing principal component analysis (PCA) using Simca 14.1 (Supplementary Fig. S2). Overall, in the PCA score plot, the distribution the 4^th^ and few 2^nd^ vaccinated samples were distinct compared to pre and 3^rd^ PBMC samples, suggesting that these samples are closely clustered according to post-vaccination time points.

### Distinct and dynamic changes in host DEGs in the high responder group

To further analyse the characteristics of DEGs related to vaccine responsiveness, up- or down-regulated DEGs were compared in the NR, LR or HR groups. The 2^nd^ vaccination showed highest number of up- or down-regulated DEGs (Supplementary Table S1), so we further compared the transcriptomic changes in the HR groups compared to the NR and LR groups. There were 327 up-regulated DEGs and 114 down-regulated DEGs in the HRs relative to the NRs & LRs (Fig. 1d). To examine the biological roles of these DEGs, a gene ontology (GO) enrichment analysis was applied to the up-regulated genes among HR vs NR & LR. GO analysis confirmed that vaccination led to enriched GO terms in lymphocyte-, B cell- and immunoglobulin-mediated immunity, B cell activation and signalling pathway and the immunoglobulin complex (Supplementary Table S2).

Furthermore, we assumed that 3 groups with very few subjects (6, 8 and 5 vaccinees in NR, LR and HR, respectively) may have over-complicated the class-comparison. So, to observe the relationships and differences between genes among HR and NR + LR, we inserted the raw expression file containing normalized levels of genes, in CEMiTool (https://cemitool.sysbio.tools) using 2 groups (HR vs LR + NR). CEMiTool can identify sets of co-expressed genes and capture relationships between genes within each module^16^. CEMiTool identified 4 different co-expression modules in two groups (Supplementary Fig. S3). Gene set enrichment analysis in Supplementary Fig. S3, showed that the module activity is different at different time points (pre, 2^nd^, 3^rd^ and 4^th^) of HR vs NR + LR for both up- and down-regulated genes. Module M1 was significantly enriched with neutrophil signalling pathways, along with T cell differentiation, as expected from an immunogenic response caused by our vaccine (Supplementary Fig. S3 and Table S3). Furthermore, module M2 was significantly enriched for phagocytosis and B cell receptor signalling pathways (Supplementary Fig. S3 and Table S4).

### Identification of upregulated immunogenic genes among high responders

To elucidate the role of gene expression, we firstly analysed those affected genes which were significantly upregulated at each dose among HR in comparison with each dose of NR + LR. The genes were selected by comparing the *p* value and FC of each gene at 2^nd^, 3^rd^, and 4^th^ vaccination among HR vs 2^nd^, 3^rd^ and 4^th^ vaccination among NR + LR. The top 10 DEGs (based on highest FC with *p* < 0.05), at 2^nd^, 3^rd^, and 4^th^ vaccination among HR compared with 2^nd^, 3^rd^ and 4^th^ vaccination among NR + LR, are given at Supplementary Tables S5–S7, respectively. Furthermore, we assumed that immune stimulant metabolic and gene markers can be identified among vaccinees that are with high antibodies titers (HR). For identification, those markers among HR, the genes upregulated at 2^nd^, 3^rd^, and 4^th^ vaccination against the baseline were extracted among HR. Top 10 upregulated DEGs in the HR group are given in Table 2. Details of all DEGs with *p* < 0.05 and FC >1 among 2^nd^, 3^rd^, and 4^th^ HR vaccinees compared with baseline is given in Supplementary Tables S8–S10, respectively. Interestingly, among top 10 DEGs, we identified DEGs involved in T lymphocytes and Natural Killer cells (NK cells) activation, such as CD69, CD83 following 2^nd^ and 3^rd^ vaccination, and CXCR4 as well as immunoglobulin-related genes, such as IGLV2–11, IGLC2, IGHG1, IGKV3D-20, and IGLV1–40, following the fourth vaccination. In addition, to see if the top 10 DEGs at Table 2 among HR may have been affected in NR + LR, we searched top 10 genes at 2^nd^, 3^rd^, and 4^th^ dose of HR (Table 2) among all significant genes of 2^nd^, 3^rd^, and 4^th^ dose of NR + LR. CANT1, CD69, CXCR4 were found significantly affected (*p* < 0.05 and FC >2) at 2^nd^ vaccination of NR + LR, CANT1, TENM4, CD69 were found significantly affected (*p* < 0.05 and FC >2) at 3^rd^ vaccination of NR + LR, and UCKL1, ALOX5, ITPK1were found significantly affected (*p* < 0.05 and FC >2) at 4^th^ vaccination of NR + LR (Supplementary Table S11). However, none of these were among the list of top 10 DEGs at 2^nd^, 3^rd^, and 4^th^ vaccination among NR + LR compared with baseline. These results suggested that top affected DEGs in Table 2 were specifically affected among HR.

**Table 2:** Top 10 up-regulated DEGs in high responders after each vaccination.

Top 10 DEG	2^nd^ vaccination	3^rd^ vaccination	4^th^ vaccination
1	CANT1	CANT1	ALPL
2	ITPK1	TENM4	IGLV2–11
3	CD69	CD69	IGLC2
4	POLR3E	POLR3E	IGKV3D-20
5	NAMPT	MS4A3	IGHG1
6	CD83	ALOX5	CANT1
7	NADSYN1	PLXNA3	UCKL1
8	CXCR4	ITPK1	POLR3E
9	ALPL	FZD3	ALOX5
10	ALOX5	ALPL	ITPK1

Further to observe if the top 10 DEGs of Table 2 correlate with PRNT_50_ and IFA response, correlation analyses between the fold-change of all genes (Table 2) and PRNT_50_ titer at each time point was performed using MetaboAnalyst 4.0. Interestingly, among top DEGs, 17 genes (12 genes in common) were positively correlated with PRNT_50_ titer, except for ALPL which was negatively correlated (Supplementary Fig. S4 and Table S12). Our analysis suggested the expression pattern of these early vaccine responses for genes involved in T lymphocytes, NK cells and immunoglobulin-related genes, correlated with the magnitude of the antibody response measured a month after each vaccination.

### Vaccination impact on metabolic profiles of vaccinees’ sera

To examine the vaccine induced metabolic alteration between the NR, LR, and HR groups, we compared the sera before the 1^st^ and after the 2^nd^, 3^rd^, and 4^th^ vaccination collectively in the three groups, by inserting apLCMS feature table containing 12,018 features into MetaboAnalyst 4.0 and SIMCA 14.1. Hierarchical clustering analysis (HCA) and partial least squares discriminant analysis (PLS-DA) were first performed Supplementary Fig. S5. Model validation with the number of permutations equalling 200 generated intercepts performed for each PLS-DA plot is given in Supplementary Fig. S6. The metabolic profile of NRs and LRs at four vaccination time points were although separated by PLS-DA (left), but however, a weak separation was observed with the unsupervised HCA (right), in Supplementary Fig. S5, suggesting a weak biological variation. In comparison, a good separation was observed among HRs, between the pre, 2^nd^, 3^rd^ and 4^th^ vaccinated sera, based on PLS-DA (left) as well as HCA (right) in Supplementary Fig. S5. This data suggest that the 2^nd^ and booster doses of vaccination was not enough for causing detectable metabolic changes in the NR and LR sera; however, both the 2^nd^ and booster (3^rd^ & 4^th^) vaccinations cause efficient metabolic changes in the serum of HR vaccinees.

**Figure 2:**
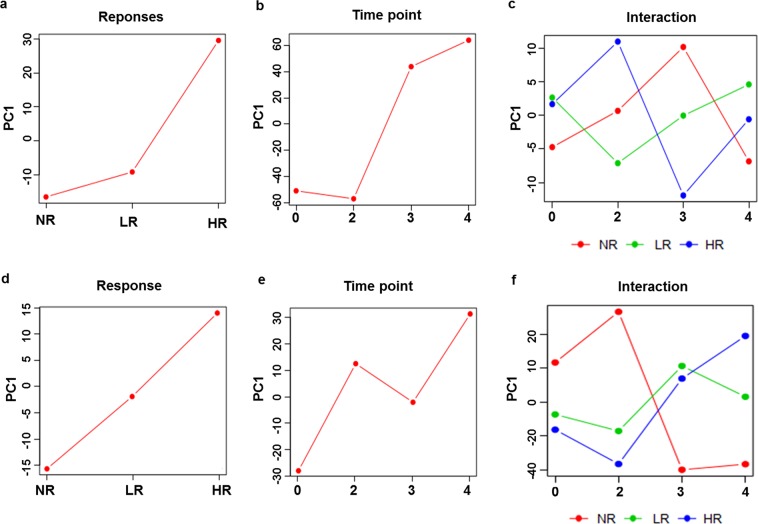
Metabolic and transcriptomic variations caused by response of vaccine and time point of each vaccination. Response of vaccine and time point of each vaccination were used as experimental factors to extract the metabolic and transcriptomics phenotype by ANOVA–simultaneous component analysis (ASCA) using *p* value threshold 0.05. (**a**–**c**) Metabolomics’ related major pattern associated with phenotype (responders), time (vaccination), and interaction between phenotype and time. ASCA result revealed 35.5%, 41.6%, and 16.8% of observed metabolic variations for the score plots of phenotype, time, and interaction, respectively. (**d**–**f**) Transcriptomics’ related major pattern associated with phenotype (responders), time (vaccination), and interaction between phenotype and time. ASCA result revealed 38.4%, 35.4%, and 14.9% of observed transcriptomics variations for the score plots of phenotype, time, and interaction, respectively. In each figure, a *p* value ≤ 0.05 was obtained with 100-permutation test which were applied to confirm the variance caused by each factor. PC1 represents principal component 1.

Moreover, to assess the differential metabolic profile caused by each vaccination instance and their responses, ANOVA-simultaneous component analysis (ASCA) was performed. ASCA can analyse complex datasets, such as metabolomics or metabolic profiling experiments that contain underlying factors such as sex difference, doses or combinations thereof^17^. In essence, ASCA uses ANOVA to decompose variation associated to experimental factors and PCA to discover principal patterns of variation within these factors. As shown in Fig. 2a and Supplementary Fig. S7, the score plots of ASCA revealed 35.5% of observed metabolic variations among NRs, LRs and HRs with 249 observed significantly differential metabolites among three groups, while 281 significantly differential features with 41.6% of variation (Fig. 2b and Supplementary Fig. S7), were observed by vaccination instance (pre-, 2^nd^, 3^rd^, and 4^th^ vaccinations). Moreover, 280 significantly differential features with 16.8% of variance could be explained by the interaction of the two experimental factors (Fig. 2c and Supplementary Fig. S7). Similarly, ASCA was performed on transcriptomics data. ASCA revealed 38.4%, 35.4% and 14.9% explained variation (Fig. 2d–f), along with 119, 279 and 171 observed significantly differential genes (Supplementary Fig. S7), among score plots of response (NR vs LR vs HR), time point (vaccination instance), and their interaction, respectively. The score plot of metabolite’s response based on principal component (PC) 1, indicates an initial increase in the score (NR to LR), followed by a sudden rise in the score (LR to HR), while that of genes showed a linear increase. This trajectory is in accordance with the biology of vaccine induced antibody response in the form of PRNT_50_ and IFA. Similarly, in the score plot of metabolite’s time point, the trajectory shows an increasing pattern in the score from 2^nd^ to 3^rd^ and 4^th^ vaccination, which was slightly different with gene’s time point plot at 2^nd^ and 3^rd^ time points. The pre vaccinated sera at 0 time point of NR, LR and HR showed no metabolic difference, especially, the LRs and HRs are merged. However, after 2^nd^, 3^rd^ and 4^th^ vaccination, the trajectory of NR, LR and HR were separated from each other with a cumulative interaction of 16.8% variation. While in gene’s interaction plot, the trajectory of LRs and HRs indifferent with NRs, moved closely to each other, except for the 4^th^ vaccinated sera, where the three groups were separated by 14.9% of explained variation (Fig. 2). These results suggest that the response to vaccination also classified the vaccinees in three groups: NRs, LRs, and HRs. In addition, the vaccination instance was highly significant and each vaccination showed differential metabolic phenomena.

**Figure 3:**
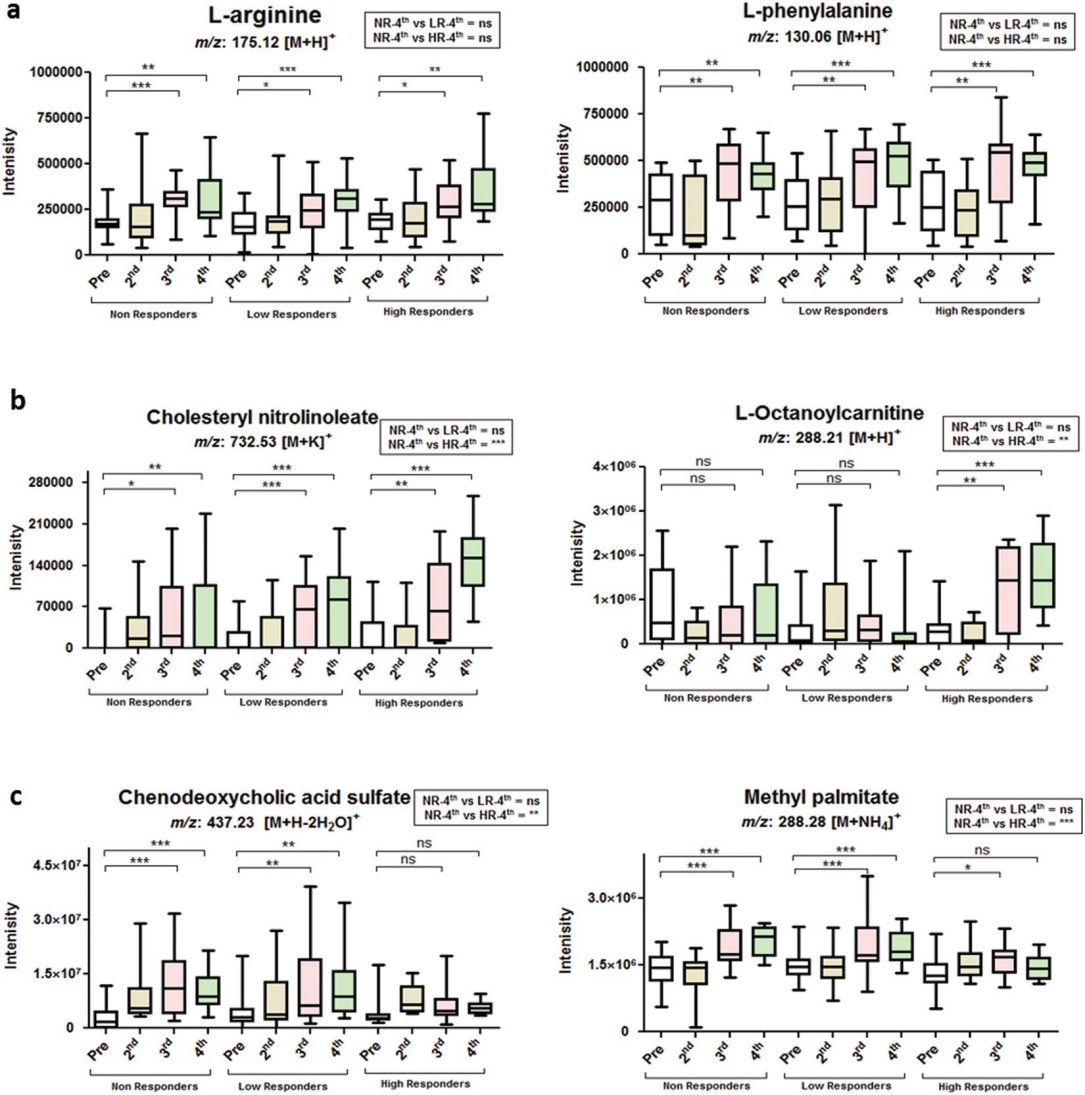
Relative concentrations of metabolic signatures in sera after vaccination. Relative concentrations of significant compounds detected in pre-vaccination sera, as well as in 2^nd^, 3^rd^, and 4^th^ post-vaccination sera, among NRs, LRs, and HRs, extracted from significant features obtained from response or time point or their interaction analysis by ASCA. (**a**) Relative concentrations of arginine (left) and phenylalanine (right) after each vaccination. (**b**) Relative concentrations of cholesteryl nitrolinoleate (left) and L-octanoylcarnitine (right) extracted from interaction of responder and vaccination instance. (**c**) Relative concentrations of chenodeoxycholic acid sulfate (left) and methyl palmitate (right) extracted from interaction of responder and vaccination instance. ***p ≤ 0.001; **p ≤ 0.01; *p ≤ 0.05; ^ns^, not significant (*p* > 0.05), per student’s *t*-test; ^NR-4th^, non-responders after 4^th^ vaccination; ^LR-4th^, low responders after 4^th^ vaccination; ^HR-4th^, high responders after 4^th^ vaccination.

To identify the metabolic features responsible for variant metabolic phenomena among NRs, LRs, and HRs caused by vaccination, we extracted the significant metabolites with high leverage and low squared prediction error (SPE), using leverage and alpha threshold of 0.9 and 0.05, respectively. (Supplementary Fig. S7 and Table S13)^18^. Afterwards, we identified those immune-related metabolic signatures which correlated with response and time point (Fig. 2a,b), irrespective of antibody response. Arginine and phenylalanine were upregulated in a dose-dependent manner among all vaccinees correlated with time points (Fig. 2c), regardless of their PRNT_50_ and IFA antibody response (Fig. 3a). Additionally, correlated to PRNT_50_ and IFA antibody titers, as well as to the interaction (Supplementary Fig. S7), among responders and vaccination instance (Fig. 2c), cholesteryl nitrolinoleate and octanoylcarnitine^19,20^, were significantly increased among HRs (Fig. 3b). While, chenodeoxycholic acid and methyl palmitate levels were elevated among NRs and LRs, but not among HRs (Fig. 3c). Such an increase in the levels of these immunomodulatory compounds in NRs and LRs might have a role in their poor response to the Hantavax vaccine^21–23^. In addition, correlated to interaction (Fig. 2c), tyrosine, N stearoyl tyrosine, 16-hydroxy palmitate, ubiquinone-9, benzoate, indole-3-acetaldehyde were detected. Tyrosine, ubiquinone-9, and benzoate were significantly high among HRs after 4^th^ vaccination compared with NRs (Supplementary Fig. S8).

### Interaction network analysis between differentially expressed genes and metabolites

**Figure 4:**
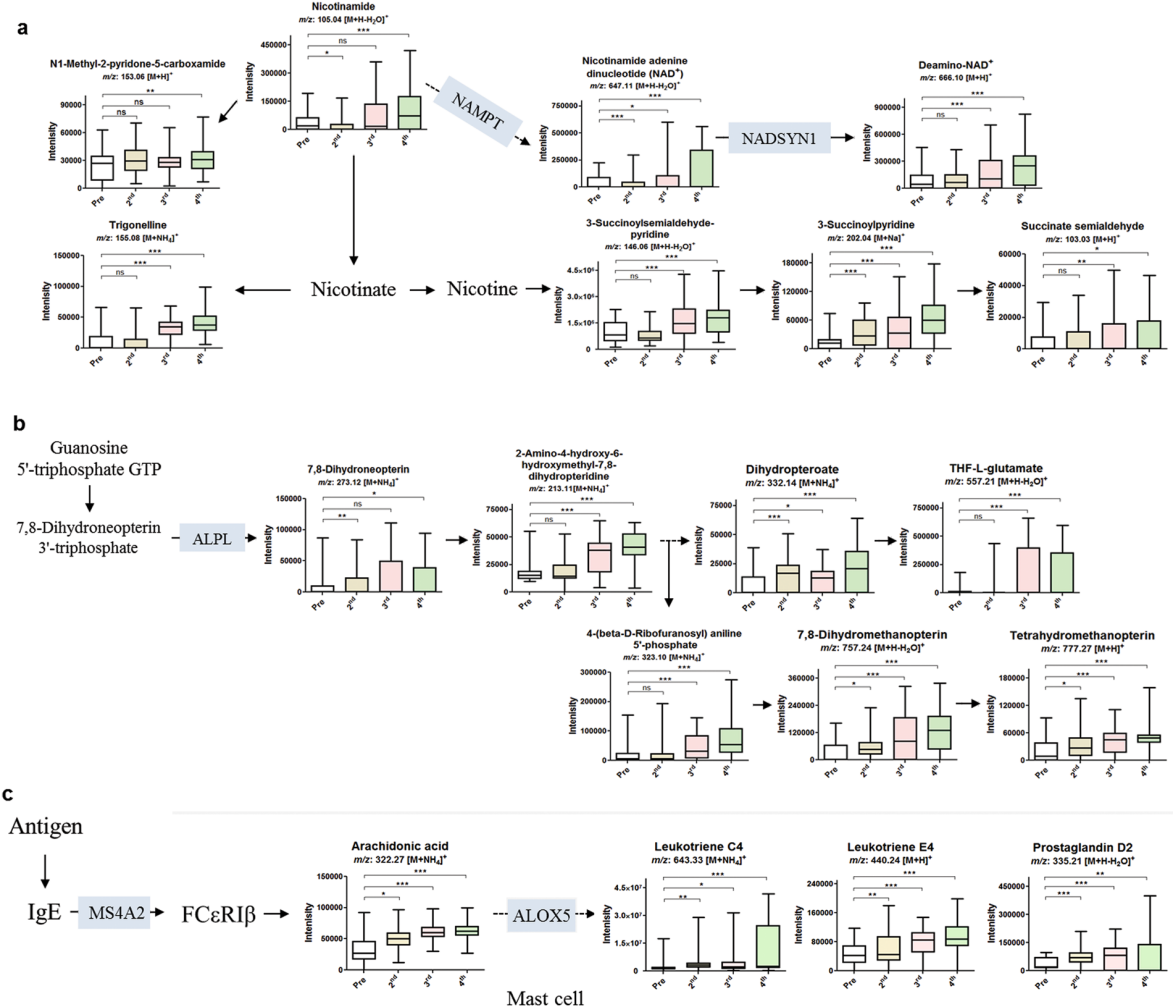
Pathway overview of nicotinate & nicotinamide metabolism, folate biosynthesis, and arachidonic acid metabolism correlated with the top DEGs. (**a**) Relative concentrations of significantly upregulated metabolites in nicotinate and nicotinamide metabolism pathways, detected before the 1^st^ and after the 2^nd^, 3^rd^, and 4^th^ vaccinations (all), correlated with the upregulated *NAMPT* and *NADSYN1* genes after the 2^nd^ vaccination. (**b**) Relative concentrations of significantly upregulated metabolites in folate biosynthesis pathway detected before the 1^st^ and after the 2^nd^, 3^rd^, and 4^th^ vaccinations (all), correlated with the upregulated *ALPL* gene after the 2^nd^ and 4^th^ vaccinations. (**c**) Relative concentrations of significantly upregulated metabolites in arachidonic acid metabolism pathway detected before the 1^st^ and after the 2^nd^, 3^rd^, and 4^th^ vaccinations (all), correlated with the upregulated *MS4A2* and *ALOX5* genes after the 3^rd^ vaccination. ***p ≤ 0.001; **p ≤ 0.01; *p ≤ 0.05; ^ns^, not significant (*p* > 0.05), per student’s *t*-test.

Gene–metabolite interaction was further assessed to validate the DEGs and to elucidate the association between the DEGs and differentially expressed metabolites, as well as to unveil the metabolic pathways affected by those DEGs. To do so, metabolites along with the genes extracted from leverage and SPE plots (Supplementary Fig. S7), of response, time point and their interaction were inserted with the KEGG ids and gene name in KEGG database. The results are given in Supplementary Fig. S9. Affected pathways through interaction of each vaccination instance among the three responders were more of our interest. Fatty acid metabolism, Th17 cell differentiation, antigen processing and presentation, NF-kappa B signalling pathway, phenylalanine metabolism, phagosome, Fc gamma R-mediated phagocytosis, biosynthesis of amino acids, RNA transport, and human T-cell leukemia virus 1 infection were highly affected by vaccination instance among the three groups (Supplementary Fig. S9). Further to perform in depth analysis, the metabolites and genes among all vaccinees that were significantly upregulated (FC >2, p-value < 0.05), including the top 30 DEGs in the vaccinees (Table 2), were screened for in the KEGG database (https://www.genome.jp/kegg). Supplementary Fig. S10 shows the interaction network analysis of genes and metabolites. These black dots representing the metabolites and red lines representing the DEGs hits in human metabolic pathway, reveals that most of the metabolites are closely connected with other metabolites as well as the DEGs. While, the bars represent the % hits of significant metabolites (blue) and genes (green) in each pathway. The following top 20 pathways were significantly enriched, namely; arachidonic acid metabolism, steroid hormone biosynthesis, 2-oxocarboxylic acid metabolism, ABC transporters, biosynthesis of amino acids, phenylalanine metabolism, tryptophan metabolism, chemical carcinogenesis, arginine and proline metabolism, tyrosine metabolism, fatty acid degradation, pyrimidine metabolism, linoleic acid metabolism, thiamine metabolism, folate biosynthesis, nicotinate and nicotinamide metabolism, histidine metabolism, human T-cell leukemia virus 1 infection, propanoate metabolism, primary bile acid biosynthesis. To check the interaction relationship, we focused on those metabolic pathways (red circled), where metabolites were affected in connection with the top 30 DEGs of Table 2. ABC transporter and chemical carcinogenesis pathways were excluded due to several KEGG ids were with similar *m/z*. *NAMPT* and *NADSYN1* genes, among the top 10 DEGs after the 2^nd^ vaccination (Table 2), were interconnected with dose-dependently upregulated metabolites of nicotinate and nicotinamide metabolism (Fig. 4a). Similarly, *ALPL* among vaccinees after the 2^nd^ and 4^th^ vaccinations (Table 2) was involved in the upregulation of folate biosynthesis metabolites (Fig. 4b). In addition, *MS4A2* among the vaccinees after the 3^rd^ vaccination and *ALOX5* among vaccinees after each vaccination (Table 2) upregulated the MAPK-signaling pathway, leading to arachidonic acid metabolism pathway’s metabolites in a dose-dependent manner among all vaccinees (Fig. 4c). Furthermore, a similar pattern of elevated dose-dependent metabolites was observed in these pathways, among NRs, LRs, and HRs, with no significance among the 4^th^ vaccinated NRs and LRs compared to the 4^th^ vaccinated HRs, an exception being trigonelline and 3-succinoylpyridine (Supplementary Fig. S11).

**Figure 5:**
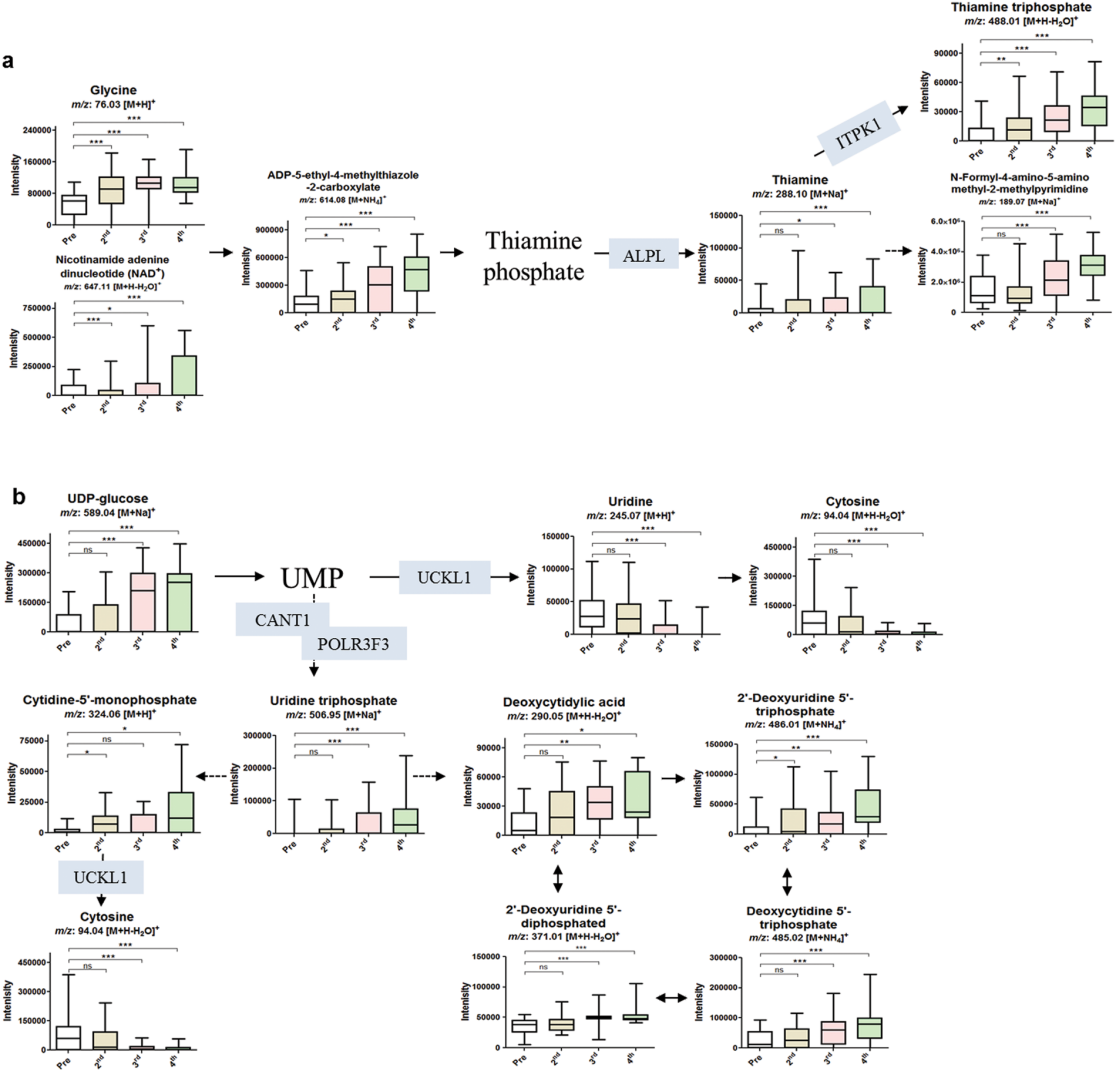
Pathway overview of thiamine metabolism and pyrimidine metabolism correlated with the top DEGs. (**a**) Relative concentrations of significantly upregulated metabolites in thiamine metabolism pathway detected before the 1^st^ and after the 2^nd^, 3^rd^, and 4^th^ vaccinations (all), correlated to the upregulated *ALPL* and *TPK1* genes after the 2^nd^, 3^rd^, and 4^th^ vaccinations. (**b**) Relative concentrations of significantly upregulated metabolites in pyrimidine metabolism pathway detected before the 1^st^ and after the 2^nd^, 3^rd^, and 4^th^ vaccinations (all), correlated to the upregulated *POLR3F3*, *CANT1*, *UCKL1*, and *POLD3* genes after the 2^nd^, 3^rd^, and 4^th^ vaccinations. ***p ≤ 0.001; **p ≤ 0.01; *p ≤ 0.05; ^ns^, not significant (*p* > 0.05), per student’s *t*-test.

Moreover, we selected thiamine metabolism and pyrimidine metabolism as DEGs (Table 2) hits in these pathways were upregulated among HR vaccinees following 2^nd^, 3^rd^, and 4^th^ vaccinations (Fig. 5). *ALPL* and *TPK1* upregulated were connected with dose-dependent increased production thiamine metabolism’s metabolites in all vaccinees (Fig. 5a), with negligible difference between NRs, LRs, and HRs (Supplementary Fig. S12). *POLRF3* and *CANT1* at all time points and *UCKL1* in 4^th^ vaccinated vaccinees were connected with upregulated pyrimidine metabolism’s metabolites in all vaccinees (Fig. 5b), with a negligible difference between NRs, LRs, and HRs (Supplementary Fig. S12). The overexpression of the genes involved in the thiamine and pyrimidine metabolism may explain the increased production of their metabolites.

### Effect of age on gene and metabolic profiles

Many studies have reported that age can affect the quantitative antibody response. As discussed, in this study, 6 NR subjects were with average age of 46.4 ± 9.7 yr, LR were 42.0 ± 10.6 yr, and HR were 31.0 ± 9.5 yr (Table 1). In-depth-individual age for each responder group is given in Supplementary Table S14, indicating that each responder group were consisting of random aged people, especially NR consisting 30 s, 40 s and 50 s aged people. However, to exclude the possibility of age as a confounding factor in our previous analysis, we analysed the raw gene expressions and raw apLCMS (metabolites) after distributing the vaccinees by age and employed PCA and PLS-DA using MetaboAnalyst 4.0. However, the score plot of PCA and PLS-DA did not separate the vaccinees according to their age, an in addition, Kruskal–Wallis one-way analysis of variance showed no significant difference in the age of NRs, LRs and HRs (Supplementary Fig. S13). Further to confirm if the age distribution may affect the upregulated genes interconnected with the pathways given in Figs 4, 5, we analysed the raw expression of *NADSYN1*, *NAMPT*, *ALPL*, *MS4A2*, *ALOX5*, *ITPK1*, *CANT1*, *UCKL1*, *POLR3F* in age distributed individual vaccinees after 4^th^ vaccination. As shown in Supplementary Fig. S13, no significant difference was observed in age distributed vaccinees. These results may evidence a weak effect of age distribution on our previous result.

## Discussion

Global emerging zoonotic pathogens are a major threat to public health worldwide. Hantavax has been used since 1990 and has led to reduction of HFRS cases in South Korea, however, its mechanism of protection is not fully determined^24^. In this study, we provide a comprehensive transcriptomic and metabolomic analysis of Hantavax-vaccinated subjects. Several biological and metabolomics pathways including B cell differentiation and antibody production were found to be involved in HR groups. Moreover, correlated with dose dependent increased PRNT_50_ and IFA antibody response, and based on interaction between the responders at each vaccination’s time point, cholesteryl nitrolinoleate and octanoyl-carnitine were significantly elevated only among HRs, while chenodeoxycholic acid and methyl palmitate were elevated among NRs and LRs. In addition, activation of β2-receptors on B cells and Th1 cell by upregulated tyrosine and ubiqionone-9, among HRs compared with NRs, perhaps have favoured their PRNT50 and IFA responses^25–27^; however, further studies are warranted.

Enrichment of neutrophil signalling pathway, T cell differentiation pathway, phagocytosis and B cell receptor signalling pathways suggested that several gene signatures were associated with immunogenic response to Hantavax immunization. There was also a close positive correlation between vaccine responsiveness and expression levels of key transcription factors that control protective immunity, including CD69 and immunoglobulin-related genes. It is also noteworthy that CD69 was highly expressed in the top 10 upregulated genes in the HR group, but not in the NR or LR groups. Previous studies demonstrate that CD69 is considered as an early activation immune marker^28–30^. Based on our data, it is possible that the expression of immune-related DEGs in the HR group from early vaccination may have facilitated an increased expression of immunoglobulin-related genes, which may have led robust production of anti-HTNV neutralizing antibodies. Additionally, Th17 cell differentiation, antigen processing and presentation, NF-kappa B signalling pathway, Fc gamma R-mediated phagocytosis, and human T-cell leukaemia virus 1 infection among the ASCA’s based affected pathways by interaction of responder and vaccination instance may predict individual and dose related variations among responders.

In addition to transcriptomic data, metabolomic analyses identified an elevated level of immune-associated metabolites such as arginine, phenylalanine, cholesteryl nitrolinoleate, and octanoylcarnitine, in the HR group. Both arginine and phenylalanine help synthesis of nitric oxide (NO) by activation of macrophages and expression of guanosine triphosphate (GTP) cyclohydrolase I^31,32^. In addition, arginine, through its effect on NO synthase, possesses an essential role for the antiviral effect against herpes simplex virus^33,34^. Here, dose-dependently upregulated arginine and phenylalanine, may predict the immune-associated NO activation by our vaccine, regardless of its high or poor antibodies response among vaccinees, as arginine and phenylalanine were upregulated among all vaccinees regardless of no, low or high response^35^. One possibility for elevated cholesteryl nitrolinoleate level in HRs might be an increased expression and/or expense of iNOS, as cholesteryl nitrolinoleate level was previously reported to increase 20-fold after macrophage activation by lipopolysaccharide plus interferon-γ and increased the expression of iNOS in *in-vitro* study^36^. Correlated to IFA antibody response, several other amino acids including histidine, valine, tryptophan, leucine, isoleucine, homocysteine, methionine, threonine, glycine, cystathionine, glutamine, and citrulline were elevated dose dependently among all vaccinees (Supplementary Fig. 14). Further study may need to consider the effect of these amino acids and their connection with iNOS activity. In addition, in future studies, the roles of tyrosine, N-stearoyl tyrosine, hydoxypalmitate, and ubiqionone-9 should be taken in to account.

Similarly, plasma levels of medium-chain acyl carnitines such as octanoyl-carnitine were reduced in HIV patients, but however, during antiretroviral therapy, its level was significantly increased, and carnitine was proposed with an important association in oxidative stress and lymphocyte apoptosis in HIV‐infected patients^19^. We observed that the elevated level cholesteryl nitrolinoleate and octanoyl-carnitine was higher in the HR group compared to NRs and LRs groups, especially after 4^th^ vaccination. These may partly have a role in the high antibodies response among HRs, because reduced propionylcarnitine and octanoylcarnitine were reported as unspecific markers of immune suppression that may influence the function of CD4 + T cells or other PBMC subsets^19^. Additionally, highly elevated chenodeoxycholic acid (a bile acid derivative) and methyl palmitate among NRs and LRs compared with the HRs, could possibly be associated with poor response among NRs and LRs vaccinees^21–23^. According to previous studies, the enhancement of such metabolites can diminish the immunogenicity of a vaccine, as chenodeoxycholic acid can inhibit antiviral activity of IFN^21^, and methyl palmitate inhibits phagocytic activity and NO production^22,23^.

In HRs, the upregulation of DEGs in association with the metabolites in metabolic pathways possibly has a dose-related response, since different DEGs were upregulated at each vaccination doses. Therefore, the capacity of the vaccine to upregulate DEGs and metabolites is possibly dependent on its induction of memory. For example, *NAMPT*, *NADSYN1*, and *MS4A2* genes were increased after the 2^nd^ and 3^rd^ vaccination, but their associated metabolites were elevated even after the 4^th^ vaccination suggesting a vaccine induced memory response. As 2^nd^ and 3^rd^ doses were administered a month apart but the 4^th^ vaccination was performed 12 months after the 3^rd^ vaccination. Similarly, several immunoglobulin-related genes, such as IGLV2–11, IGLC2, IGHG1, IGKV3D-20, and IGLV1–40 were although highly affected only among 4^th^ vaccinated HRs, but the metabolites associated with these DEGs were increased in a dose-dependent manner, suggesting that these DEGs (likewise metabolites) might have been produced dose-dependently, but a silenced response. Such genes response favors the long term immunogenicity of Hantavax, as *NAMPT* is important for lymphocyte survival, while NAD-depleting agents are associated with the death of lymphocytes^37^, and its inhibition may cause macrophages malfunctioning^38^. Our vaccinees showed elevated level of NAD- associated metabolites and increased expression of *NAMPT* and *NADSYN1*^39^. Moreover, folate biosynthesis metabolites in our vaccinees might have caused the activation of cell-mediated immune activity by Hantavax^40^, as evidenced in enrichment analysis. Similarly, upregulated arachidonic acid metabolites are associated with immune regulation (e.g. mast cells)^41,42^. Our study, in accordance with several previous ones, provides positive correlations between gene-metabolite associations for the immunogenic response that occurred with the vaccination. In addition, these genes correlated the antibodies responses which were measured a month after each vaccination. However, contrary to key mechanisms described in previous studies, we did not test interleukins, phagocyte activity and neutrophil recruitment, underlining the importance of validations for the roles of identified metabolites.

Interestingly, thiamine and pyrimidine metabolism, along with their associated genes, showed a dose-dependent response among vaccinees. Thiamine, through its role in expression of immunoglobulins and intracellular adhesion molecule proteins, is involved in the expression of immunoglobulins and in CD40L-mediated immunity, and the release of cytokines, chemokines, and growth factors, as well as in providing an antioxidant environment for the macrophages^43^. Thiamine metabolites elevation may further explain the enrichment of T cell differentiation and phagocytosis pathways. Additionally, in pyrimidine metabolism several metabolites were elevated associated with elevated *POLR3F* and *CANT1* expression in all vaccinees, an exception being uridine. Elevated metabolites of pyrimidine have critical roles in immune responses such as cell-mediated immunity and T cell activation for producing IL-2, IL-3, and GM-CSF^44,45^, and ultimately in overcoming bacterial or fungal infections^46^. It should be noted that our metabolomics and transcriptomics studies were performed in different biological samples, however, in support of antibody response to our vaccine, the altered metabolites and transcripts associated with pyrimidine metabolism are most likely associated with the robust immune response. These modulations may explain the vaccine efficacy based on metabolomic data coupled with transcriptomic data; however, it is compulsory to validate our findings though additional experiments.

The limitation of our study includes its modest sample size in each group along with high variation in age of each vaccine, and therefore, validations should be carried out on a larger population. It should be noted that age might have affected the quantitative IFA antibody response, as there is a potential age dependency from NR to HR, but effect of age on PRNT_50_ is doubtful^47^. Second, transcriptomic changes may have resulted from new induction of gene expression or may have simply reflected the changing cellular composition of the PBMC compartments. Given that it is impossible to obtain cellular or tissue samples from lymph nodes or spleens of healthy human subjects post vaccination, our analysis was performed with PBMC, therefore, investigations are required to determine the transcriptome in sorted cell subsets, such as B, T, NK, macrophages, and dendritic cells. In addition, metabolome profiles are originated from serum, so the associations between metabolites and transcription levels is complex, as the differing biologic samples may have dampened the ability to replicate, Nevertheless, we still found that many genes and metabolites affected by same antigen among same individuals, are directly associated via their relations in the metabolic network. We believe that our results are reliable and crucially, some of the main results should be replicated in an independent study.

Finally, this study characterized both the transcriptome and metabolome of Hantavax-vaccinated subjects. These findings provide a framework for future studies examining the molecular mechanisms by which Hantavax promotes immune protection. In summary, this study’s findings illustrate the potential for transcriptomics and untargeted metabolomics to identify genes and metabolites involved in immune responses which may propose a targeted vaccine design in future.

## Methods

### Ethics statement

A phase III, multi-center, open-labelled clinical trial was under-taken to evaluate the immunogenicity of an inactivated hantavirus vaccine (Hantavax: GreenCross Corporation, Yongin, ROK) in healthy adults aged 19–75 years (ClinicalTrials.gov Identifier: NCT02360514). The study protocol was approved by the Institutional Review Board of Korea University Guro hospital (approval number: KUGH15142) and conducted in compliance with ICH-GCP E6 guideline. All participants gave written informed consent prior to initiation of study and were required to be able to comply with all study procedures. Twenty healthy adults participated in this study, but one subject was dropped after 1^st^ vaccination. The remaining 19 individuals received four doses of Hantavax at different intervals during 2015–2017. The method used to essay PRNT_50_ and IFA is described in our previous study^14^.

### Sample preparation

Blood was drawn from each subject into EDTA-K2 Vacutainer tubes (BD Bioscience, Bedford, MA) on the day of the 1^st^ vaccination, but before the vaccination itself, and 72 hours after the 2^nd^, 3^rd^, and 4^th^ vaccination. PBMCs were isolated from these blood samples using standard Ficoll-Paque (GE Healthcare, Little Chalfont UK) with density gradient centrifugation and were processed for the RNA-sequencing procedure. Serum and plasma samples were also collected for further analyses.

### RNA-seq library construction and transcriptome sequencing

RNA quality was assessed by analysis of rRNA band integrity on an Agilent RNA 6000 Nano Kit (Agilent Technologies, Santa Clara, CA). Prior to cDNA library construction, poly (A) mRNA was enriched using 2 µg of total RNA and magnetic beads with oligo (dT) primers. The purified mRNAs were then disrupted into short fragments, and double-stranded cDNAs were immediately synthesized. The cDNAs were subjected to end-repair, poly (A) addition, and connection with sequencing adapters using the TruSeq RNA Sample Prep Kit (Illumina, San Diego, CA). Suitable fragments, automatically purified using a BluePippin 2% agarose gel cassette (Sage Science, Beverly, MA), were selected as templates for PCR amplification. The final library sizes and qualities were evaluated electrophoretically using an Agilent High Sensitivity DNA Kit (Agilent Technologies, Santa Clara, CA), and the fragments were found to be between 350 and 450 bp. Subsequently, the library was sequenced using an Illumina HiSeq. 2500 sequencer (Illumina, San Diego, CA). The data discussed in this publication have been deposited in NCBI’s Gene Expression Omnibus^48^, and are accessible through GEO Series accession number GSE120115. (https://www.ncbi.nlm.nih.gov/geo/query/acc.cgi?acc = GSE120115).

### Transcriptome data analysis

Low-quality reads were filtered out according to the following criteria: (i) reads containing more than 10% of skipped bases, (ii) reads containing more than 40% of bases with quality scores less than 20, and (iii) reads with average quality scores of each read of less than 20. The whole filtering process was performed using in-house scripts. Filtered reads were mapped to the mouse reference genome (Ensembl release 77), using the aligner STAR v.2.3.0e^49^. Gene expression level was measured with Cufflinks v2.1.1 using the gene annotation database of Ensemble release 77^50^. Non-coding regions genes were removed with the mask option. To improve the accuracy of measurement, multi-read-correction and frag bias-correct options were applied. All other options were set to default values. For differential expression analysis, gene-level count data were generated using the HTSeq-count v0.5.4p3 tool with the options “-m intersection-nonempty” and “–r option considering paired-end sequence”^51^. Based on the calculated read count data, DEGs were identified using the R package called TCC 1.20.1^52^. The TCC package applies robust normalisation strategies to compare tag count data. Normalization factors were calculated using the iterative DEGES/edgeR method. The *q*-value was calculated based on the *p*-value using the *p*-adjust function of the R package with default parameter settings. DEGs were identified based on a false discovery rate (FDR) *q*-value threshold of less than 0.05. Genes were considered differentially expressed when having a change of more than two-fold, with a *p*-value of less than or equal to 0.05, for at least one time point.

### GO and KEGG enrichment analysis

In order to examine the biological significance of the differentially expressed genes, we performed GO and KEGG enrichment analysis to investigate their functional and pathway annotation^53^. This analysis was performed by the following web-based functional annotation tools; DAVID v6.7 software^54^, CEMiTool^16^, and Enrichr^55^. In DAVID the differentially expressed genes and all the expressed genes were submitted as the gene list and background list, respectively. CEMiTool employed for Co-expression analysis and gene modules analysis. For CEMiTool, the raw gene expression file categorized as HR and NR + LR was inserted to see the relationships and differences between genes among groups. The modules obtained in CEMiTool were further processed for (enrichment analysis) among two groups using GO biological process 2018 in Enrichr web tool.

### Chemicals and reagents used for metabolomics analysis

High-performance liquid chromatography (HPLC)-grade water was purchased from from J.T. Baker (Phillipsburg, NJ) and acetonitrile from Tedia (Fair Lawn, NJ). Formic acid was purchased from Fluka (St. Louis, MO). All chemicals and reagents were stored at appropriate temperatures and conditions. Standard solutions and serum samples were stored at −80 °C.

### Sample preparation for metabolite extraction

Sample preparation were performed as discussed previously^56^. Briefly 50 µL aliquots of sera were first treated with 195 µL of acetonitrile and 5 μL of a mixture of 3 stable isotope standards ([3-methyl-13C]-caffeine, [dimethyl-D6]-N,N-diethyl-M-toluamide, and [13C5, 15 N]-L-methionine) (1:4, v/v). The sample extract was vigorously vortexed for 1 min, and centrifuged at 13,000 rpm at 4 °C for 10 min for protein precipitation and metabolite extraction. The supernatants containing the polar metabolites were collected for LC–MS/MS analysis.

### Analysis of metabolites by LC–MS/MS

The metabolomics profiling was performed using Ultra Performance Liquid Chromatography system (Agilent 1260 Infinity Quaternary) coupled with an Agilent LC-MS/MS Q-TOF 6550 mass spectrometer^57^. Hypersil Gold C-18 (100 × 2.1 mm) 1.9 µm analytical columns (ThermoFisher, Waltham, MA) were used to perform the analyses. The column and autosampler temperature were maintained at 45 °C and 10 °C, respectively. Solvent A (0.1% formic acid in water) and solvent B (0.1% formic acid in acetonitrile) were used as mobile phases. To detect the analytes, an electrospray ionization detector was operated with a curtain gas of 35 psi, gas temperature of 250 °C, supplied at 14 mL/min, and sheath gas temperature of 250 °C, supplied at a flow rate of 11 mL/min. All samples were run in triplicates, and data for each ionization technique were acquired in positive ion mode^58^.

### Metabolic profiling

Multivariate and univariate analyses were performed to identify molecular features that discriminated NRs, LRs, and HRs. The apLCMS provided 12,018 *m/z* (mass/charge ratio) within a range of ions set from 50 to 1,000 from mass spectral data. The data from triplicate run were averaged and statistically analysed using SIMCA 14.1 (Umetrics AB, Umea°, Sweden) and MetaboAnalyst 4.0. Unsupervised principal component analysis (PCA), supervised partial least-squares discriminant analysis (PLS-DA) was performed using SIMCA 14.1 using unit variance (UV) scaling, while unsupervised hierarchical clustering analysis (HCA) was performed using MetaboAnalyst 4.0. For HCA data sets were quantile-normalized, log-transformed, and pareto-scaled. PLS-DA, and HCA analyses were performed as follows: sera before the 1^st^ and after the 2^nd^, 3^rd^, and 4^th^ vaccinations were analyzed in NRs, LRs and HRs collectively. To ensure the quality of the PLS-DA models and to avoid the risk of over-fitting, 7-fold cross-validation (CV) was applied with 6 principal components, as 7-fold cross-validation is the default SIMCA cross-validation procedure. Two parameters: R2 (goodness of fit) and Q2 (goodness of prediction) were evaluated for each PLS-DA model. The performance of PLS-DA models was also validated by a permutation test (200 times). In addition, ASCA was performed to identify the major patterns associated with each factor (responders or vaccination instance) using MetaboAnalyst 4.0. To do so, NRs, LRs, and HRs were compared in order to identify the role of no response or low or high response as experimental factors, while, to identify the role of vaccination as a factor, sera from before the 1^st^ and after the 2^nd^, 3^rd^, and 4^th^ vaccinations were analyzed in the entire population. ASCA’s score plots were generated based on component 1. ASCA leverage and score plots were generated using the default parameters supplied by the website (www.metaboanalyst.ca), i-e the leverage threshold and alpha threshold were set to be 0.9 and 0.05, respectively. Correlation analysis was performed using MetaboAnalyst 4.0, by inserting a table containing the fold change (FC) calculated for each gene at each time point with the titer at each time point.

### Biomarkers identification

Compounds with significant changes between the two factors from ASCA’s interaction (*p*-value < 0.05) were subsequently considered important for identification as potential biomarkers related to metabolic effects caused by the vaccination. Accurate masses of potential metabolites were searched against the online biochemical database service Metlin Mass Spectrometry Database METLIN (http://metlin.scripps.edu). The recorded KEGG numbers were subjected to human metabolomics pathway (KEGG) (www.kegg.jp/kegg/kegg1.html) and MetaboAnalyst 4.0, for further enrichment analyses.

### Statistical analysis using GraphPad

Putative identities were analyzed using the GraphPad Prism software (v. 5.03; La Jolla, CA) for measurement of their relative intensities in each group. Data are presented as means ± standard deviation (SD) and differences with *p*-values < 0.05 were considered statistically significant.

## Supplementary information

Supplementary figure 1–14

Supplementary tables 1–14
