# Supplementary material for: A Systems Vaccinology Approach Reveals the Mechanisms of Immunogenic Responses to Hantavax Vaccination in Humans
Source: Sci Rep. 2019 Mar 18;9:4760. doi: 10.1038/s41598-019-41205-1 (PMC6423257; doi:10.1038/s41598-019-41205-1)
Supplement: Supplementary file 1 — Supplementary figure 1–14 [file 41598_2019_41205_MOESM1_ESM.docx]

**A Systems Vaccinology Approach Reveals the Mechanisms of Immunogenic Responses to Hantavax Vaccination in Humans**

Authors:

Adnan Khan^1#^, Ok Sarah Shin^2#^, Jinhyuk Na^1^, Jae Kwan Kim^1^, Rak-Kyun Seong^2^, Man-Seong Park^3^, Ji Yun Noh^4^, Joon Young Song^4^, Hee Jin Cheong^4^, and Youngja H Park^1*^, Woo Joo Kim^2,4*^

Author affiliations:

^1^Metabolomics Laboratory, Korea University College of Pharmacy, Sejeong city, Republic of Korea

^2^Department of Biomedical Sciences, Korea University College of Medicine, Seoul, Republic of Korea

^3^Department of Microbiology, Korea University College of Medicine, Seoul, Republic of Korea

^4^Division of Infectious Diseases, Department of Internal Medicine, Korea University College of Medicine, Seoul, Republic of Korea.

^#^These authors contributed equally to work

^*^Co-corresponding authors

Woo Joo Kim, MD., PhD., Division of Infectious Diseases, Department of Internal Medicine, Guro Hospital, Korea University College of Medicine, Gurodong-ro 148, Guro-gu, Seoul 08308, Republic of Korea. Tel: 82-2-2626-3051, Fax: 82-2-2626-1105, E-mail: wjkim@korea.ac.kr

Youngja H Park, PhD., Metabolomics Laboratory, Korea University College of Pharmacy, Sejong-ro 2511, Sejong city 30019, Republic of Korea. Tel: 82-44-860-1621, Fax: 82-44-860-1606, E-mail: [yjhwang@korea.ac.kr](mailto:yjhwang@korea.ac.kr)

Running title: Systems vaccinology analysis of Hantavax

**Supplementary fig S1.**

**
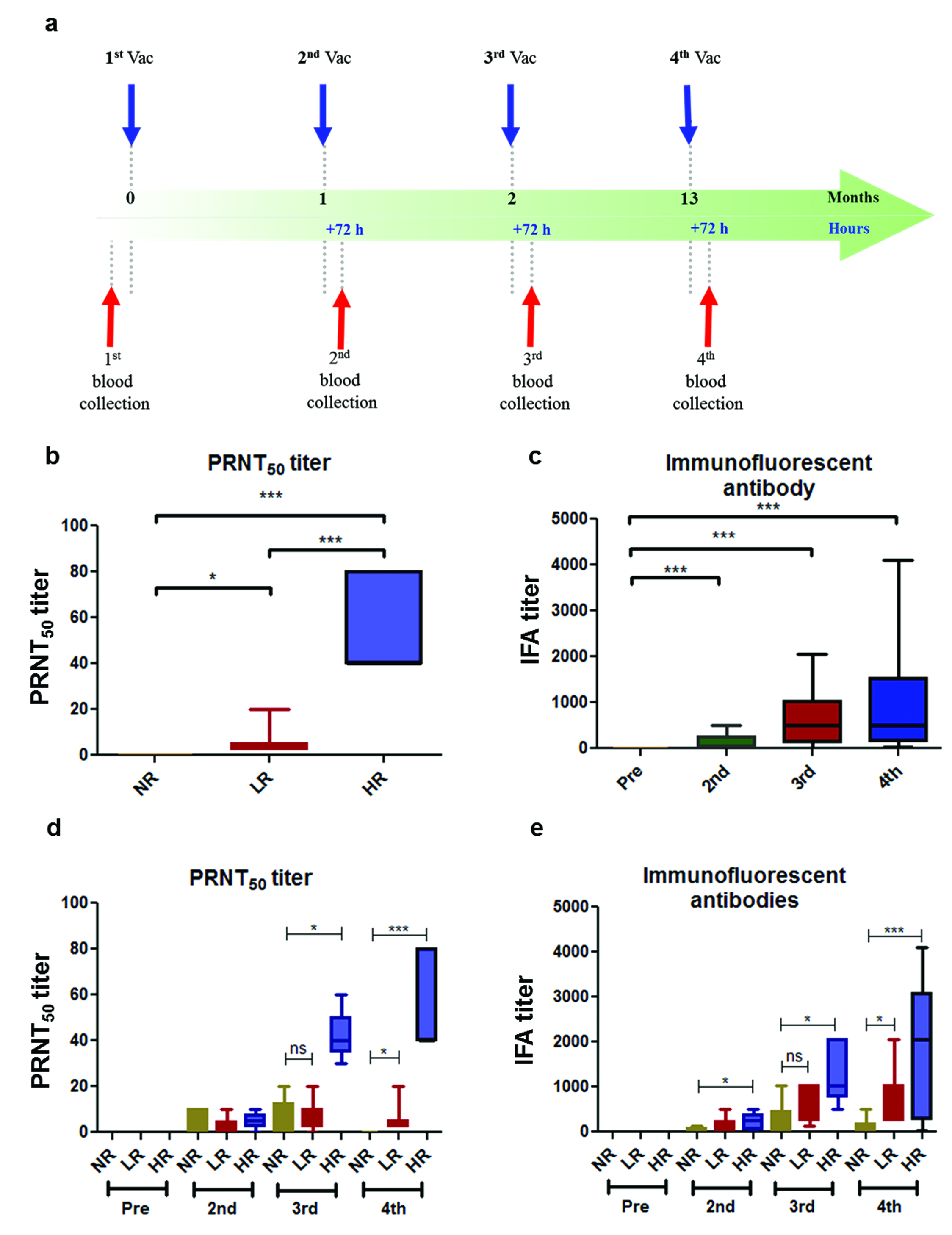
**

**Time schedule for immunization with Hantavax vaccine and antibody response:** (a) Twenty subjects were recruited and were given Hantavax four times over 13 month period. PBMCs were collected at pre-vaccination, and within 72 hours post vaccination (2^nd^, 3^rd^, and 4^th^). RNA-seq and metabolomics were performed with total 80 samples and analysed in two different ways; vaccination time and vaccine responsiveness. (b) Immunological response in the form of PRNT_50_ following Hantavax administration. Neutralizing antibody represents the PRNT_50_ titer represents the reciprocal serum dilution that reduced virus plaque number by 50%. (c) Dose stratified changes in seropositive rate immunofluorescent antibodies (IFA) after each doses over the study period. (d) PRNT_50_ titer after each vaccination among NR, LR and HR. (e) IFA after each vaccination among NR, LR and HR. ***p ≤ 0.001; **p ≤ 0.01; *p ≤ 0.05; ^ns^, not significant (p > 0.05), per student’s t-test. NR, non-responders; LR, low responders; HR, high responders.

**Supplementary fig S2.**


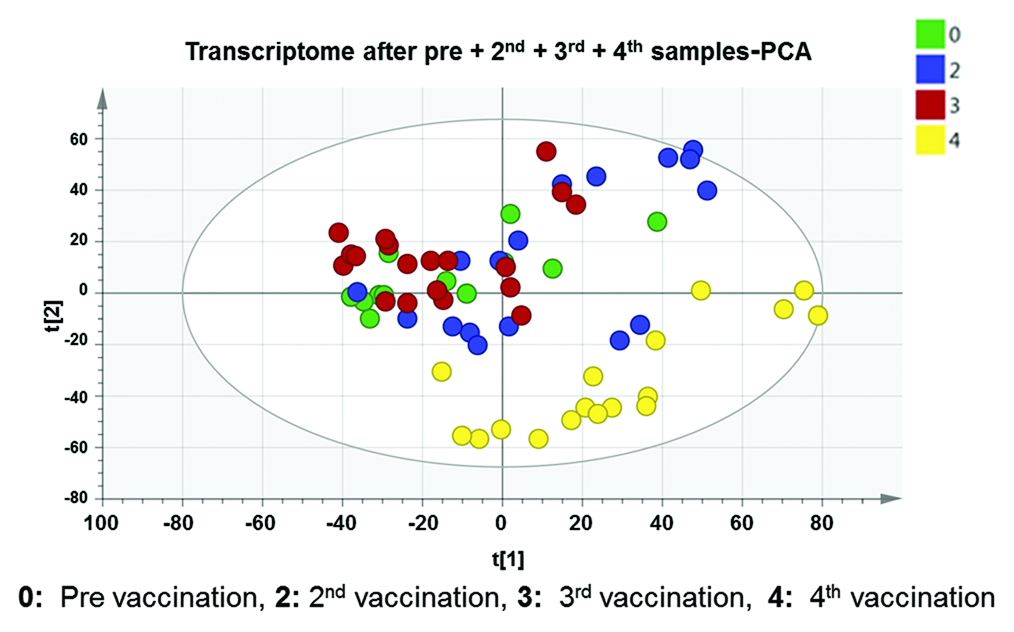


**Transcptome analysis of pre, 2^nd^, 3^rd^, and 4^th^ vaccinated sera by PCA**. Principal component analysis (PCA) between the global transcriptome-wide RNA-seq profiles of the samples. 0 represents pre-vaccinated, 2 represents 2^nd^ vaccinated 3 represents 3^rd^ vaccinated and 4 represents 4^th^ vaccinated samples. Variance explained by component 1 & 2 were 20.1 % & 14.4 % respectively.

**Supplementary fig S3.**


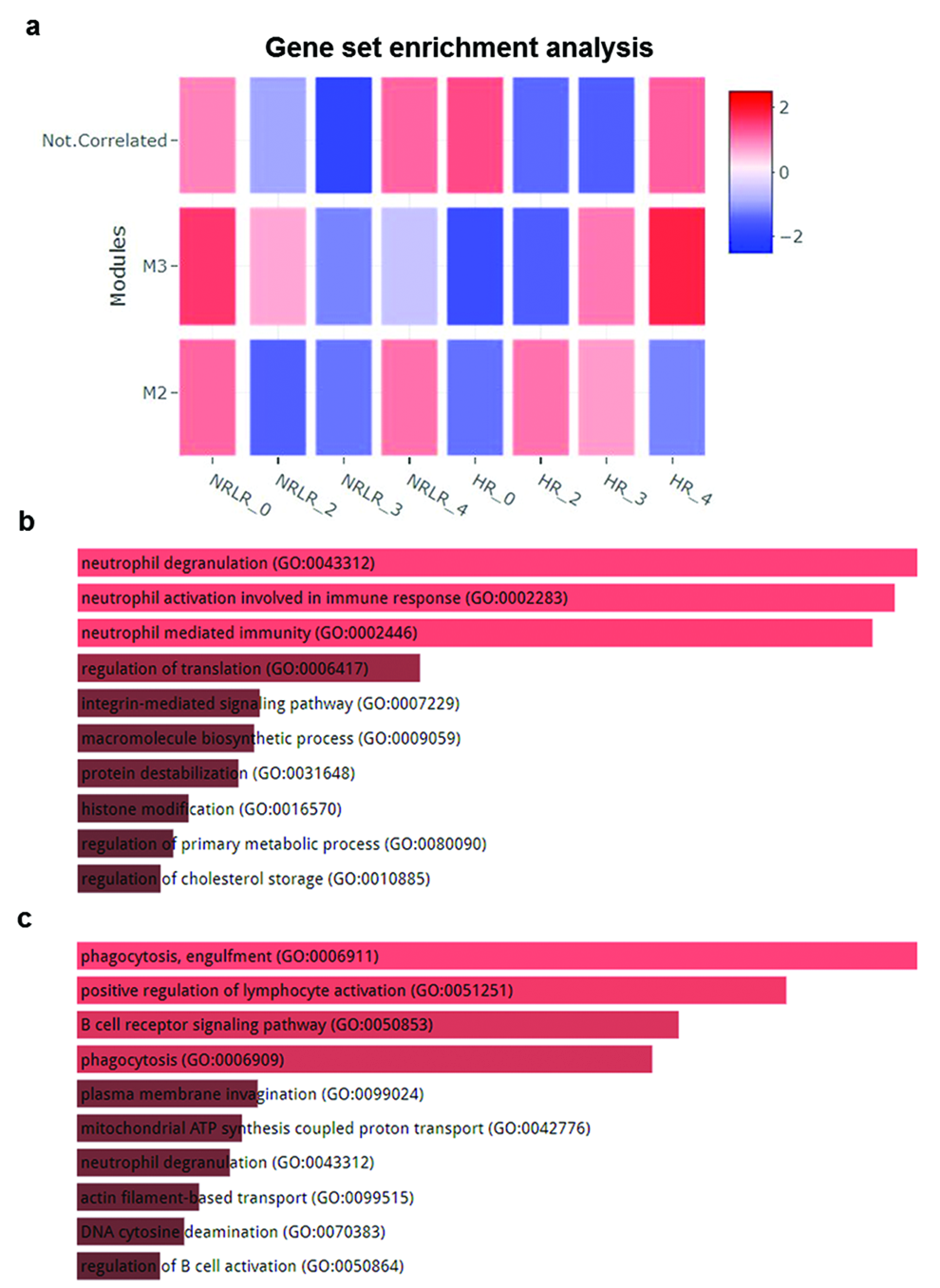


**Co-expressed gene module and GO enrichment analysis between high responders vs none- and low responders.** CEMiTool applied to HR vs NR+LR using raw gene expression. (a) Heatmap showing modules activity on each time point (0; pre vaccination, 2; 2^nd^ vaccination, 3; 3^rd^ vaccination, and 4; 4^th^ vaccinatinon) for HR and NR+LR groups. Color code normalized enrichment score of modules (red represents higher activity and blue represents lower activity). (b) Enrichr (GO biological process) analysis of the genes associated with module M1 of CEMiTool. (c) Enrichr (GO biological process) analysis of the genes associated with module M2 of CEMiTool. Boxplots showing the ten biological pathways (sorted by *p* value and z score), that are most significantly correlated and anti-correlated in modules M1 and M2 between HR and NR+LR.

**
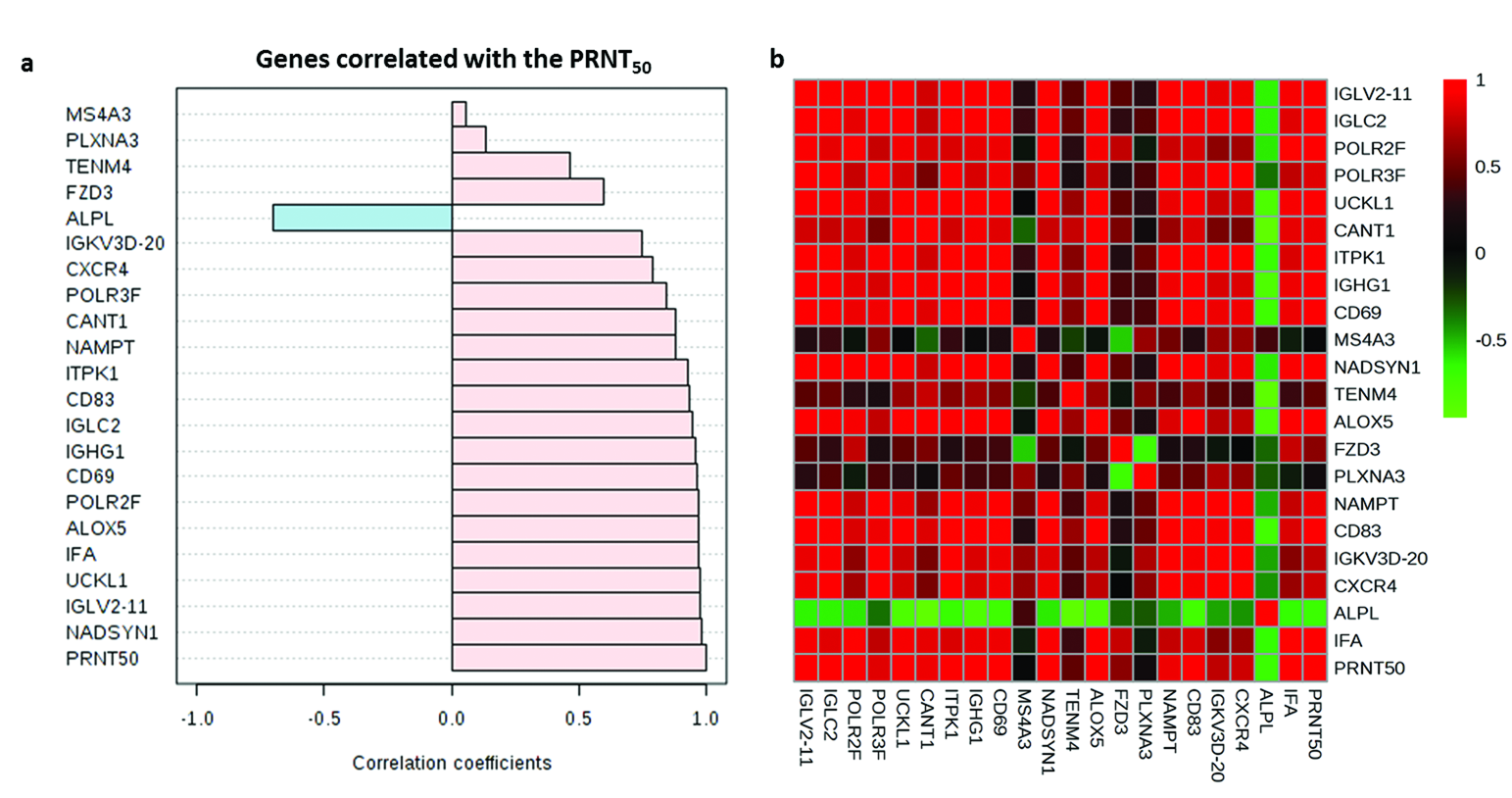
**

**Supplementary fig S4.**

**Correlations between top 10 DEGs of high reponders and PRNT_50_ antibody titer.** (a) Correlation plot showing DEGS (table 2) at each dose associated with PRNT_50_. The genes are represented as horizontal bars, with light brown indicating positive correlations with PRNT_50_ and light blue indicating negative correlations. (b) Shows the overall correlation heatmap. The red color indicates the positively correlated genes with PRNT_50_, whereas the green color represents the negative correlation. IFA represents the immunofluorescent antibody.

**Supplementary fig S5.**

**
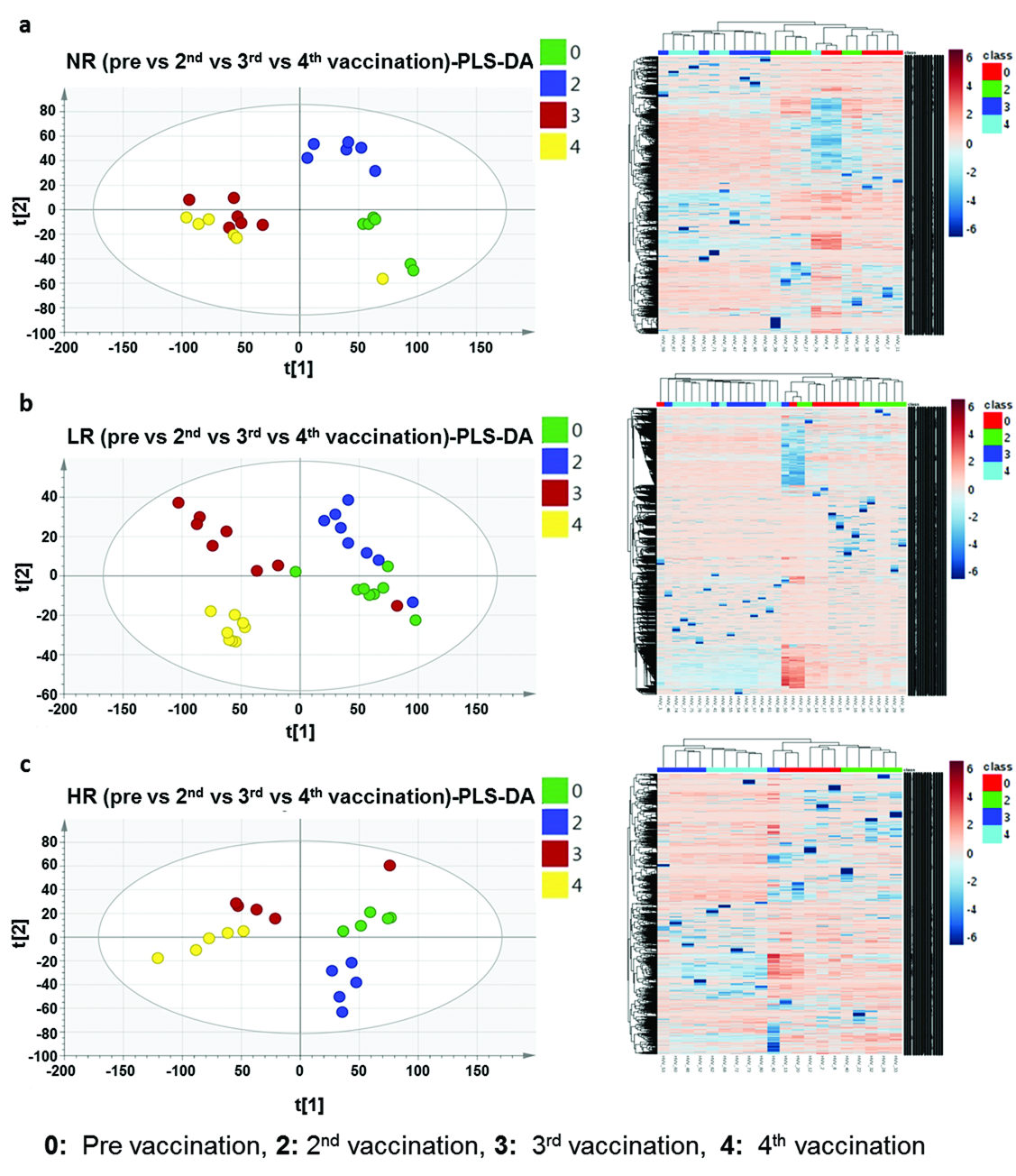
**

**Differential metabolic profiles in sera obtained from non, low and high responders.** Separation and classification of the metabolites among pre. 2^nd^, 3^rd^ and 4^th^ vaccinated sera obtained from NRs, LRs and HRs. (a) Discrimination of detected features between pre. 2^nd^, 3^rd^ and 4^th^ vaccinated sera obtained from NRs by PLS-DA (left; with 6 PCs, R^2^Y(CUM) = 0.98, Q^2^(CUM) = 0.65), and HCA (right). (b) Discrimination of detected features between pre. 2^nd^, 3^rd^ and 4^th^ vaccinated sera obtained from LRs by PLS-DA (left; with 6 PCs, R^2^Y(CUM) = 0.90, Q^2^(CUM) = 0.79), and HCA (right). (c) Discrimination of detected features between pre. 2^nd^, 3^rd^ and 4^th^ vaccinated sera obtained from HRs by PLS-DA (left; with 6PCs, R^2^Y(CUM) = 0.98, Q^2^(CUM) = 0.84), and HCA (right). In PLS-DA score plot, each data point represents one sera sample (average of triplicate run). The distance between points in the plot indicates the similarity between samples. NRs represents non responders, LRs represents low responders and HRs represents high responders.

**
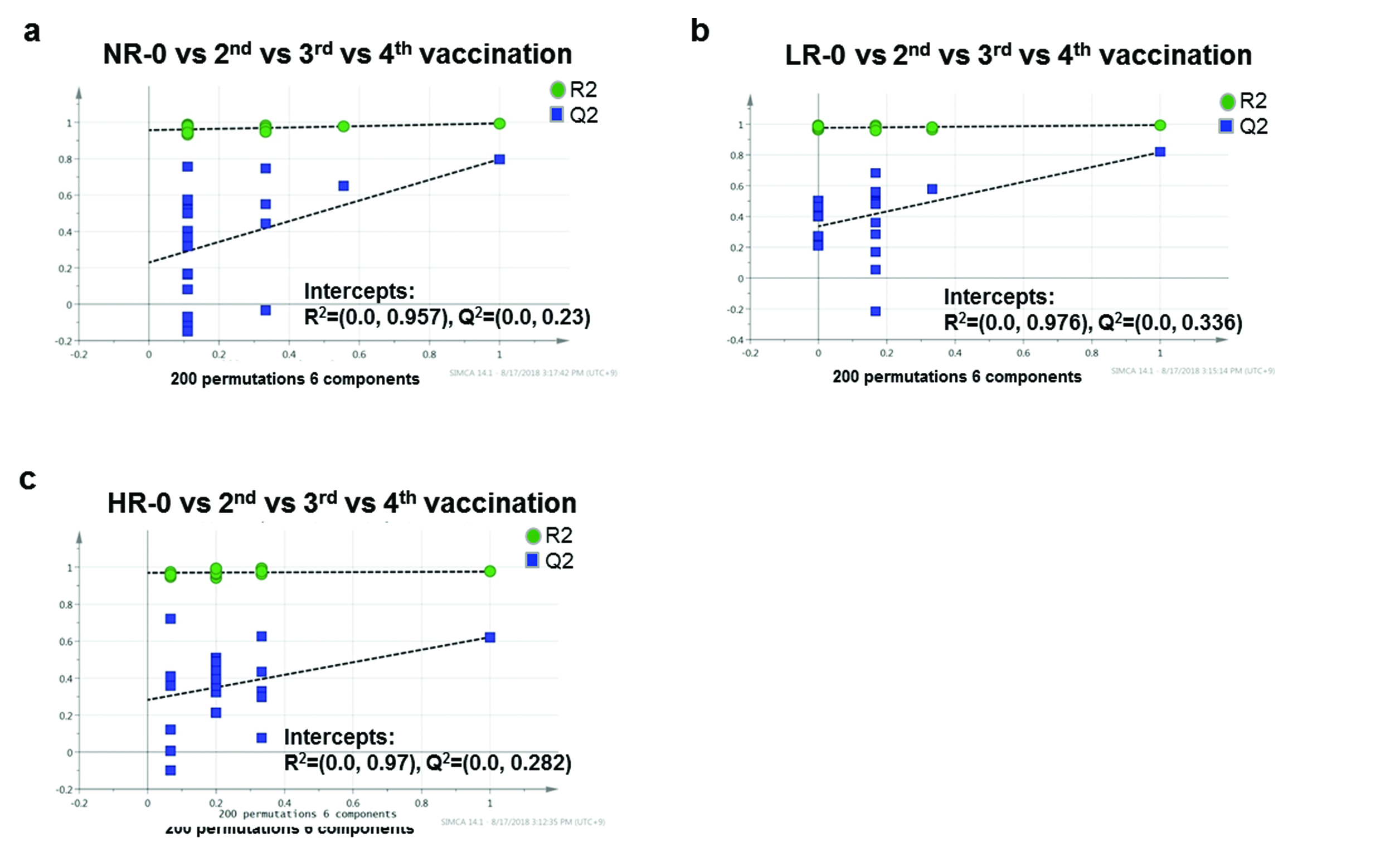
**

**Supplementary fig S6.**

**Plots of the permutation test of PLS-DA**. Demonstration of the validity of the PLS-DA model using a 200-permutation test of the six components for (a) score plot of PLS-DA for pre. 2^nd^, 3^rd^ and 4^th^ vaccinated sera obtained from NRs. Intercepts: R^2^ = 0.957 (green circles) and Q^2^ = 0.23 (blue squares). (b) pre. 2^nd^, 3^rd^ and 4^th^ vaccinated sera obtained from LRs. Intercepts: R^2^ = 0.976 (green circles) and Q^2^ = 0.336 (blue squares). (c) pre. 2^nd^, 3^rd^ and 4^th^ vaccinated sera obtained from HRs. Intercepts: R^2^ = 0.97 (green circles) and Q^2^ = 0.282 (blue squares).

**
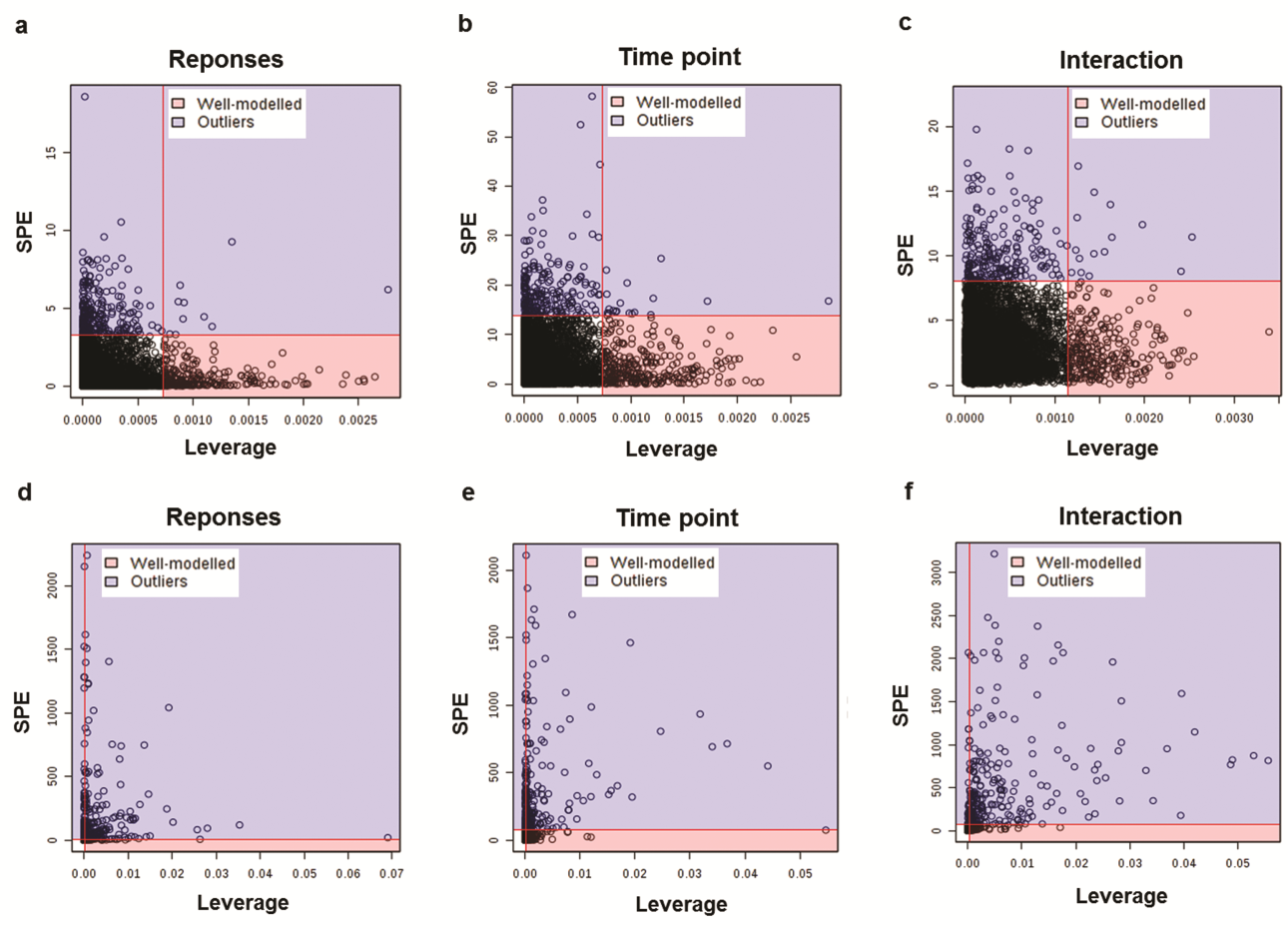
Supplementary fig S7.**

**Metabolic variations caused by response of vaccine and time point of each vaccination.** Leverage and squared prediction error (SPE) plots for ANOVA–simultaneous component analysis (ASCA) generated using leverage threshold and alpha threshold of 0.9 and 0.05, respectively. (a-c) The ASCA (for metabolomics data) selected 249, 281, and 280 important metabolites (light pink area), associated with phenotype (responders), time (vaccination), and interaction between phenotype (responders) and time (vaccination) by Leverage/SPE analysis. (d-f) The ASCA (for transcriptomics data) selected 119, 279, and 171 important genes (light pink area), associated with phenotype (responders), time (vaccination), and interaction between phenotype (responders) and time (vaccination) by Leverage/SPE analysis. NRs represents non responders, LRs represents low responders and HRs represents High responders

**Supplementary fig S8.**

**
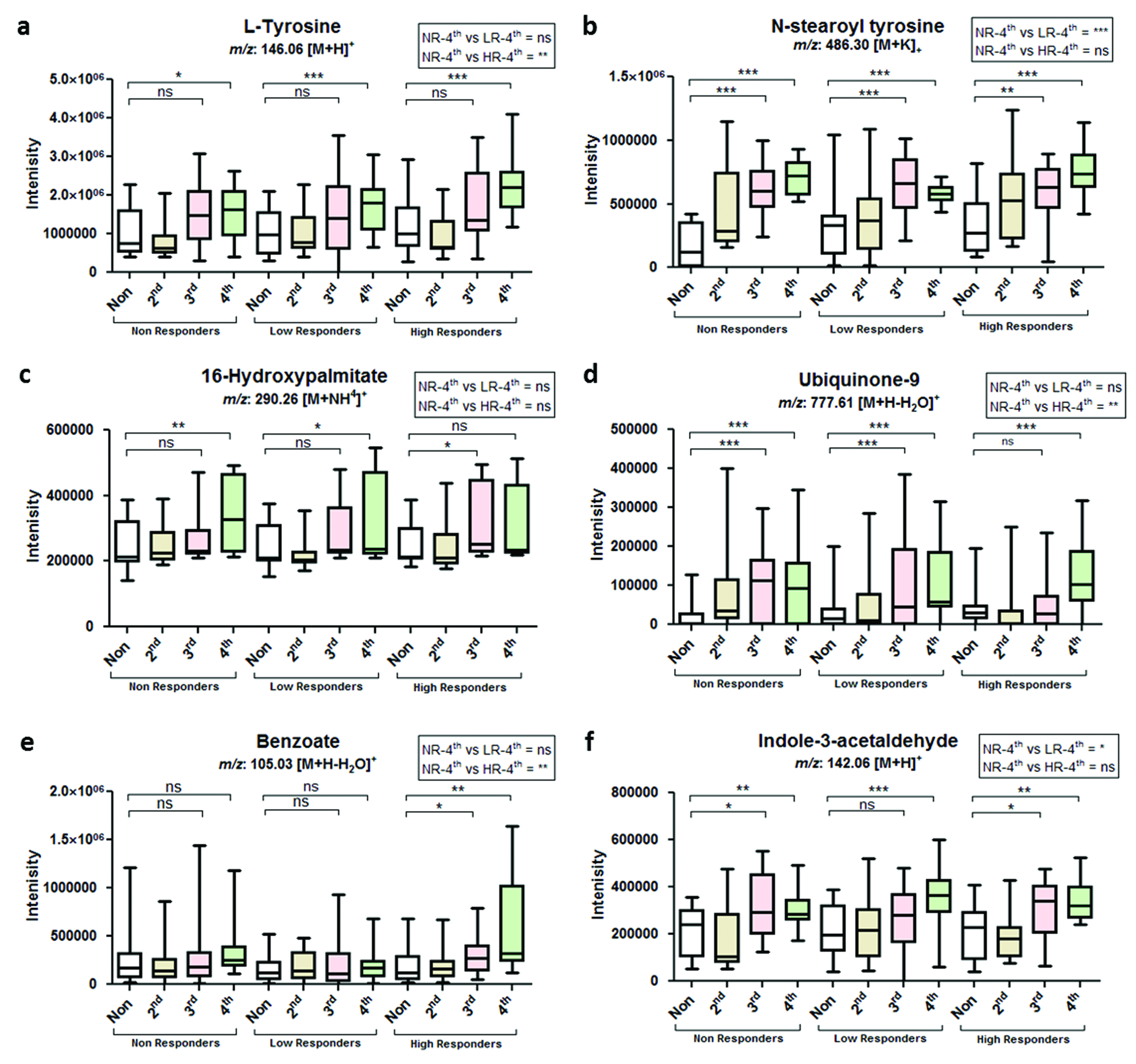
**

**Relative concentrations of metabolic signatures in sera after vaccination.** (a-f) Relative concentrations of tyrosine, N stearoyl tyrosine, 16-hydroxypalmitate, ubiquinone-9, benzoate, and indole-3-acetaldehyde detected in pre-, 2^nd^, 3^rd^, and 4^th^ post-vaccination sera, among NRs, LRs, and HRs, extracted from significant features obtained from interaction of response and time point analysis by ASCA. ****p* ≤ 0.001; ***p* ≤ 0.01; **p* ≤ 0.05; ^ns^, not significant (*p* > 0.05), per student’s *t*-test; ^NR‑4th^, non-responders after 4^th^ vaccination; ^LR‑4th^, low responders after 4^th^ vaccination; ^HR‑4th^, high responders after 4^th^ vaccination.

**Supplementary fig S9.**

**Mapping of interconnected genes and metabolites obtained from leverage and SPE plots of response, time points and interaction.** The bars represent hits (in %age) of significant metabolites and genes for (a) response (b) time point and (c) interaction in KEGG database (www.kegg.jp/kegg/kegg1.html). Each figure shows top 10 affected pathways. The blue bar represents the metabolites hits, while green are the genes hits. **
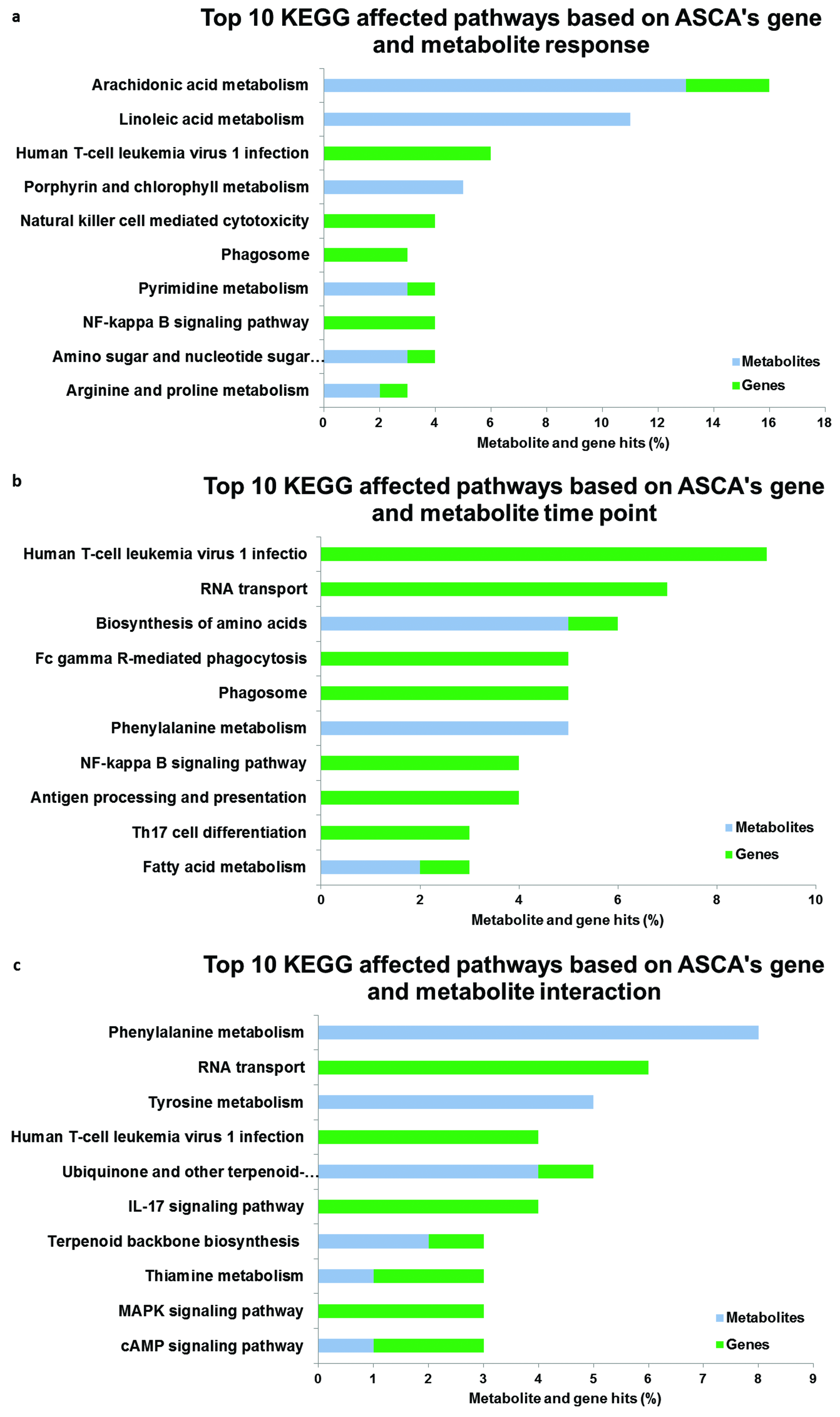
**

**Supplementary fig S10.**

**
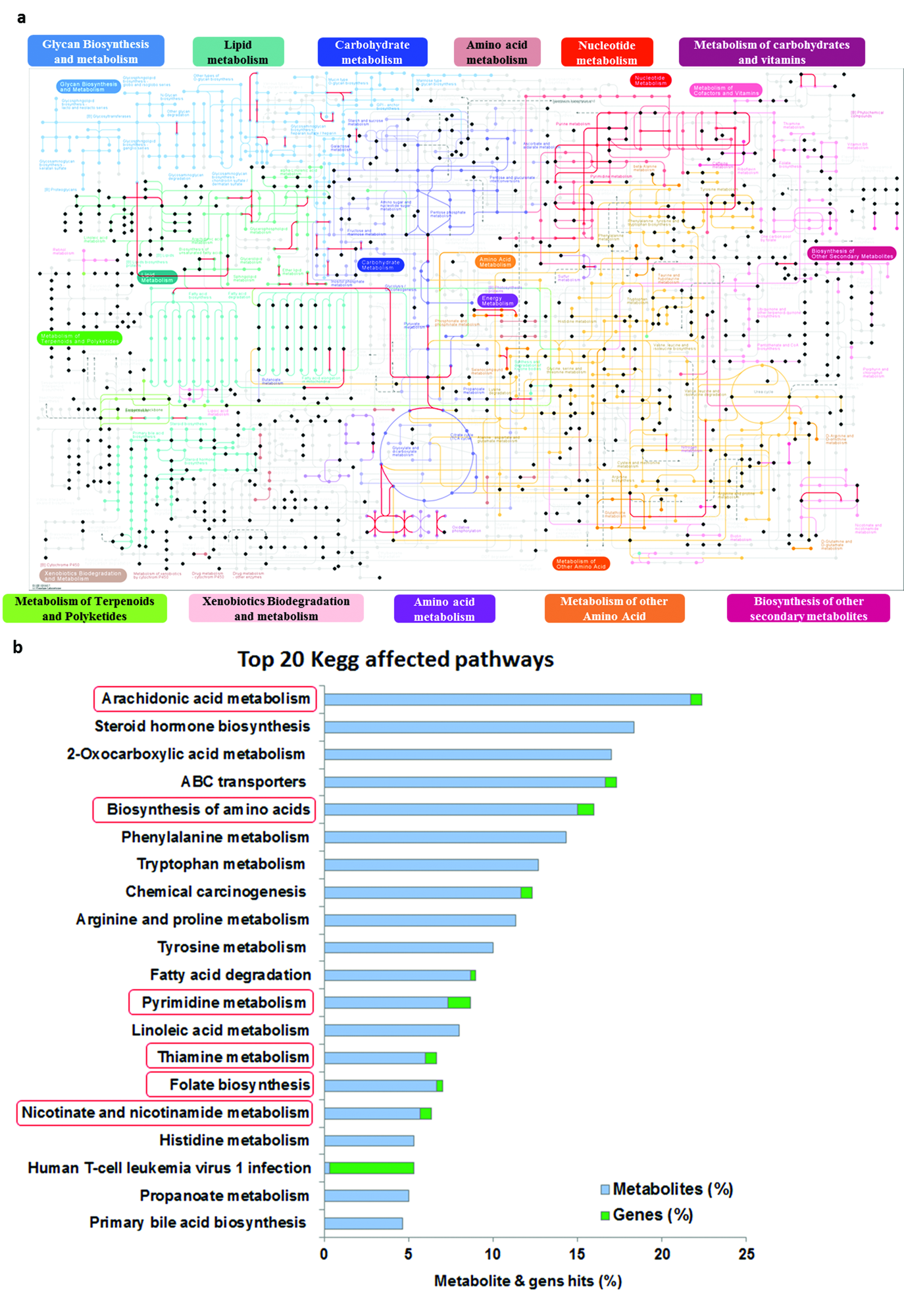
**

**Kyoto Encyclopedia Genes and Genomics (KEGG) pathway in vaccinees.** (a) Mapping of interconnected genes and metabolites. This image is obtained from KEGG (www.kegg.jp/kegg/kegg1.html). The black dots are the significant metabolites while the red lines represent the genes elevated among high responders. (b) The bars represent hits (in %age) of significant metabolites and genes in top 20 affected pathways in KEGG database. The blue bar represents the metabolites hits, while green are the genes hits. Red circled pathways represent those pathways where metabolites were affected in connection with the top 30 DEGs.

**
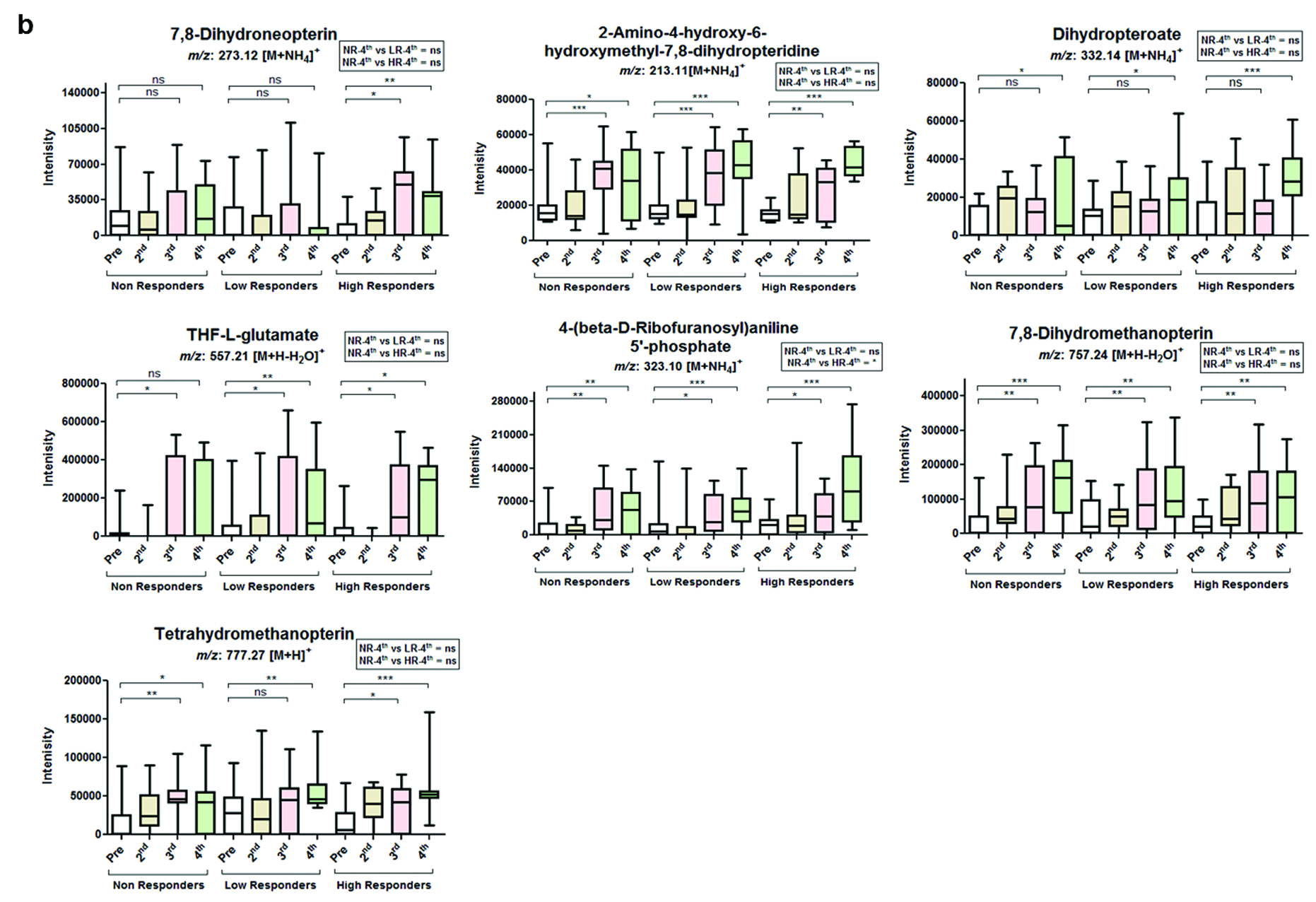

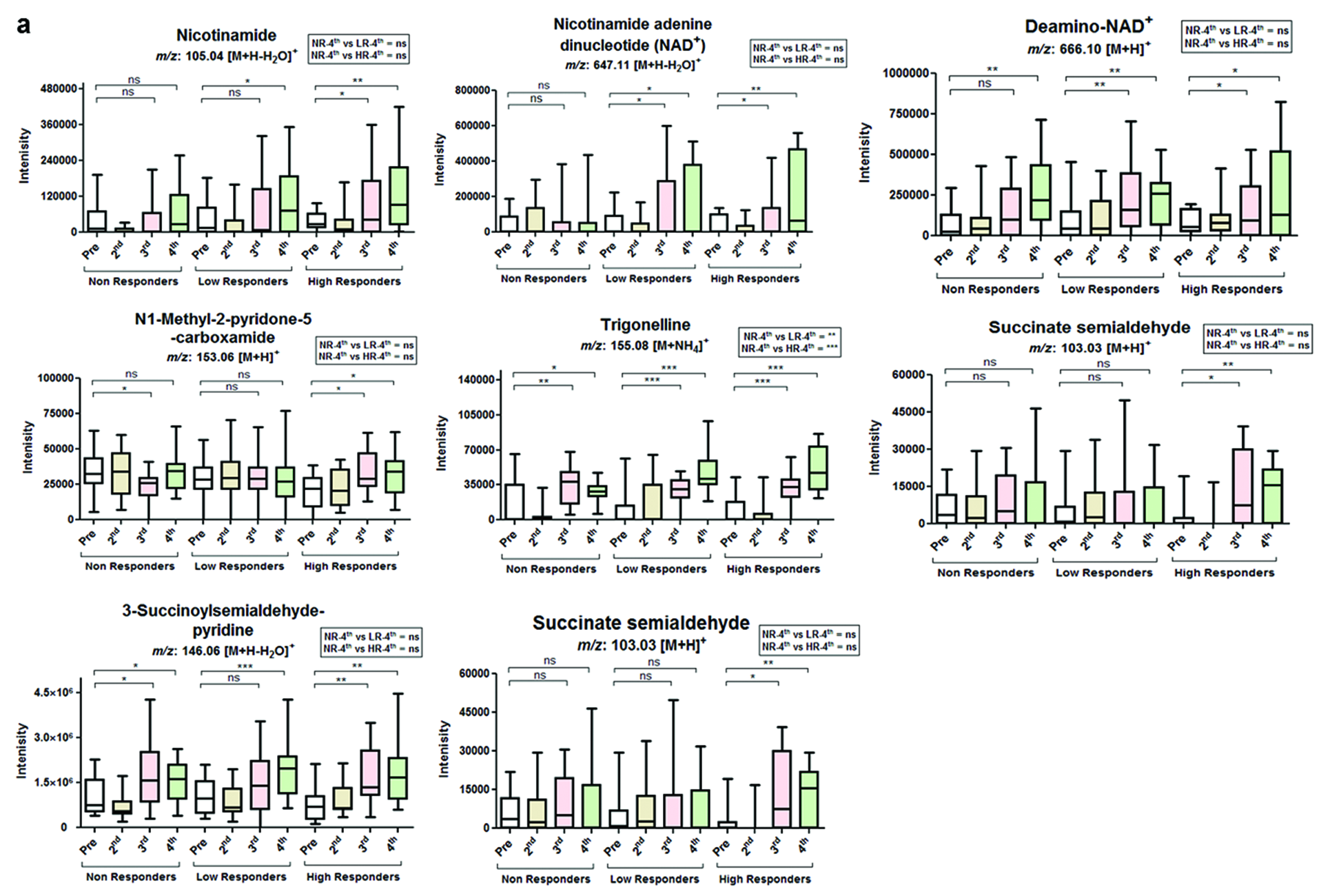
Supplementary fig S11.**

**
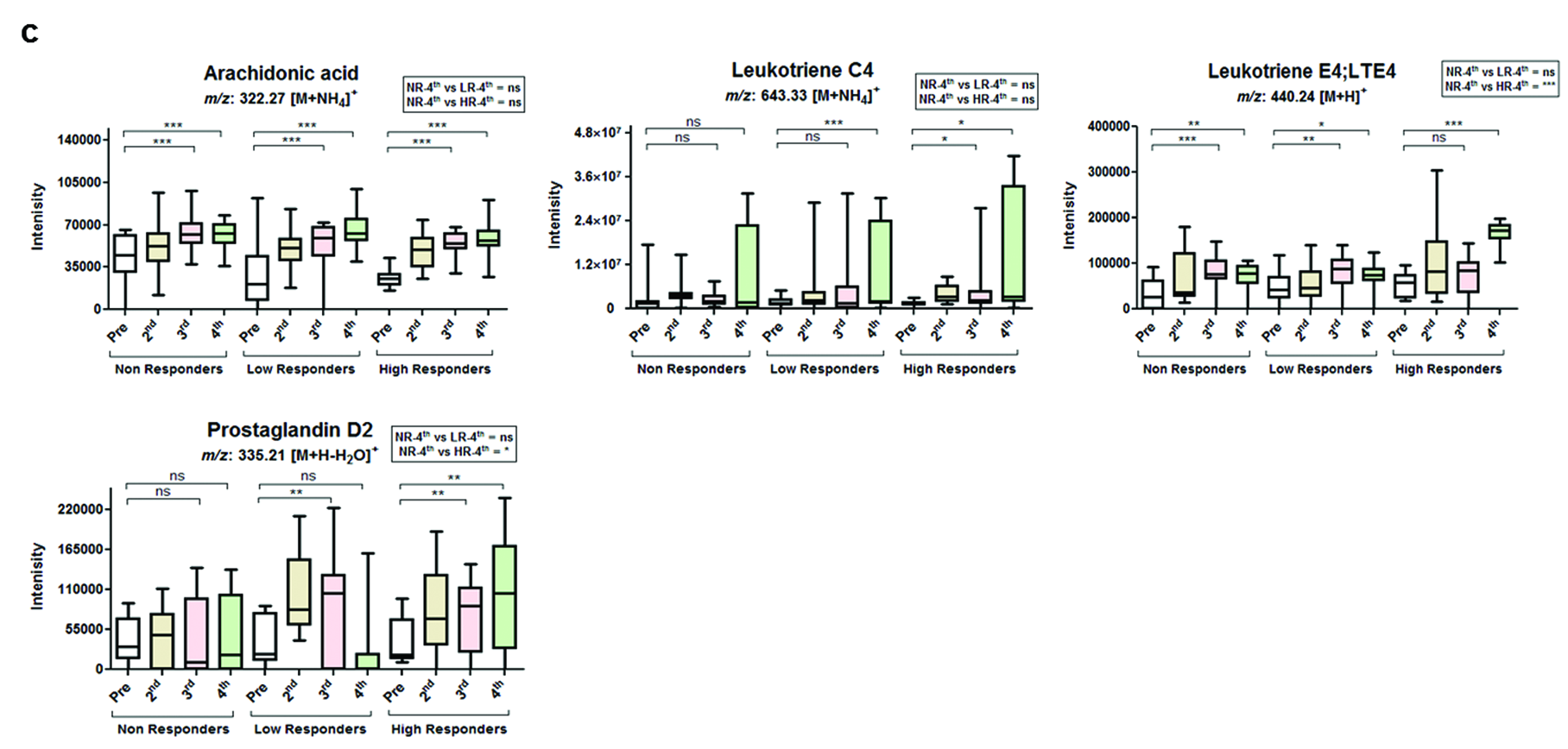
**

**Separately analyzed nicotinate and nicotinamide metabolism, folate biosynthesis** **and arachidonic acid metabolism pathway’s metabolites in none, low and high responders.** (a) Relative concentrations of significant upregulated metabolites in nicotinate and nicotinamide metabolism pathway detected in pre, 2^nd^, 3^rd^, and 4^th^, analyzed separately among NRs, LRs, and HRs vaccinees. (b) Relative concentrations of significant upregulated metabolites in folate biosynthesis pathways detected in pre, 2^nd^, 3^rd^, and 4^th^, analyzed separately among NRs, LRs, and HRs vaccinees. (c) Relative concentrations of significant upregulated metabolites in arachidonic acid metabolism pathway detected in pre, 2^nd^, 3^rd^, and 4^th^ vaccinees, analyzed separately among NR or LR or HR vaccinees. ****p* ≤ 0.001; ***p* ≤ 0.01; **p* ≤ 0.05; ^ns^not significant (*p* > 0.05) per student’s t-test, ^NR-4th^ non responders-4^th^ vaccinated,^L-4th^ low responders-4^th^ vaccinated , ^HR-4th^ high responders-4^th^ vaccinated.

**
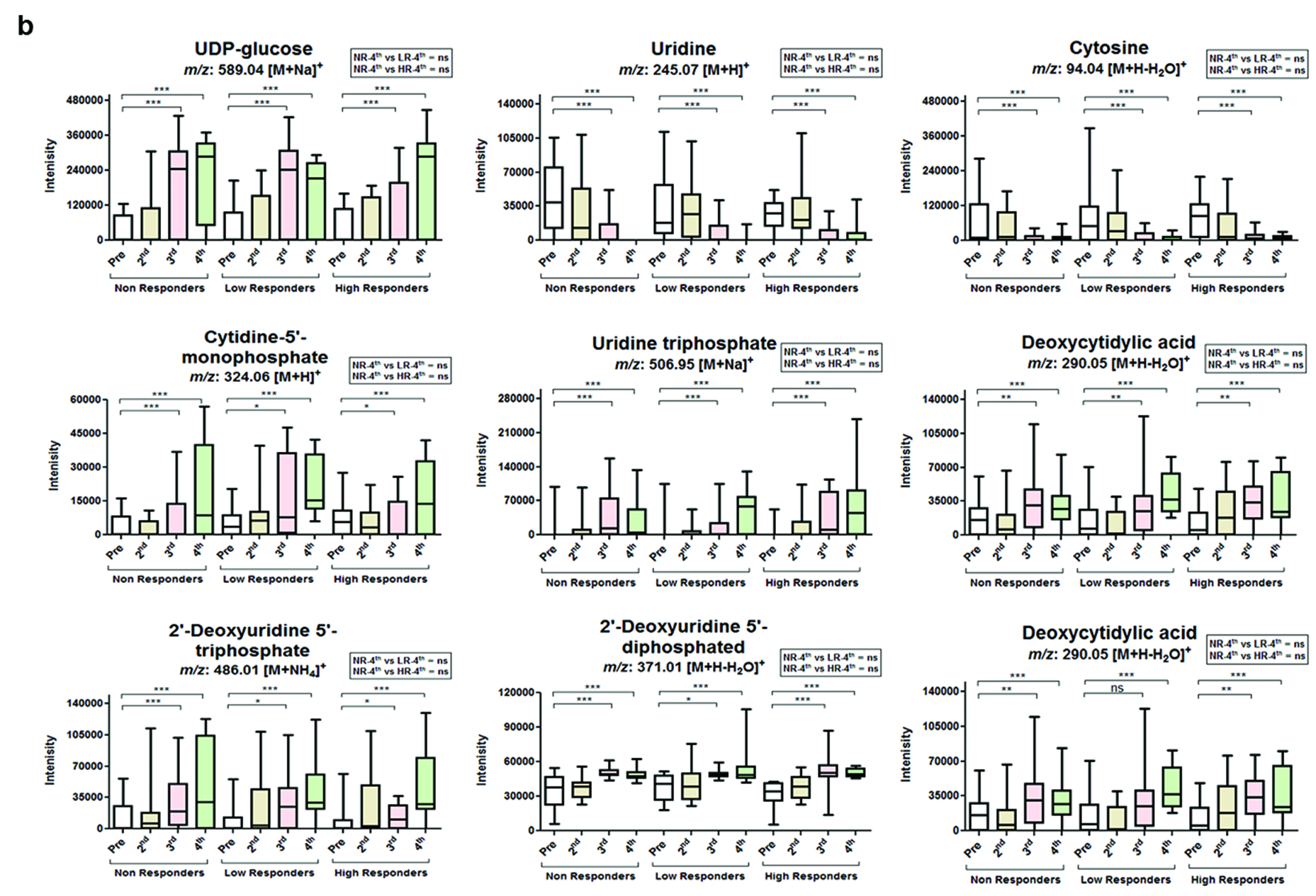

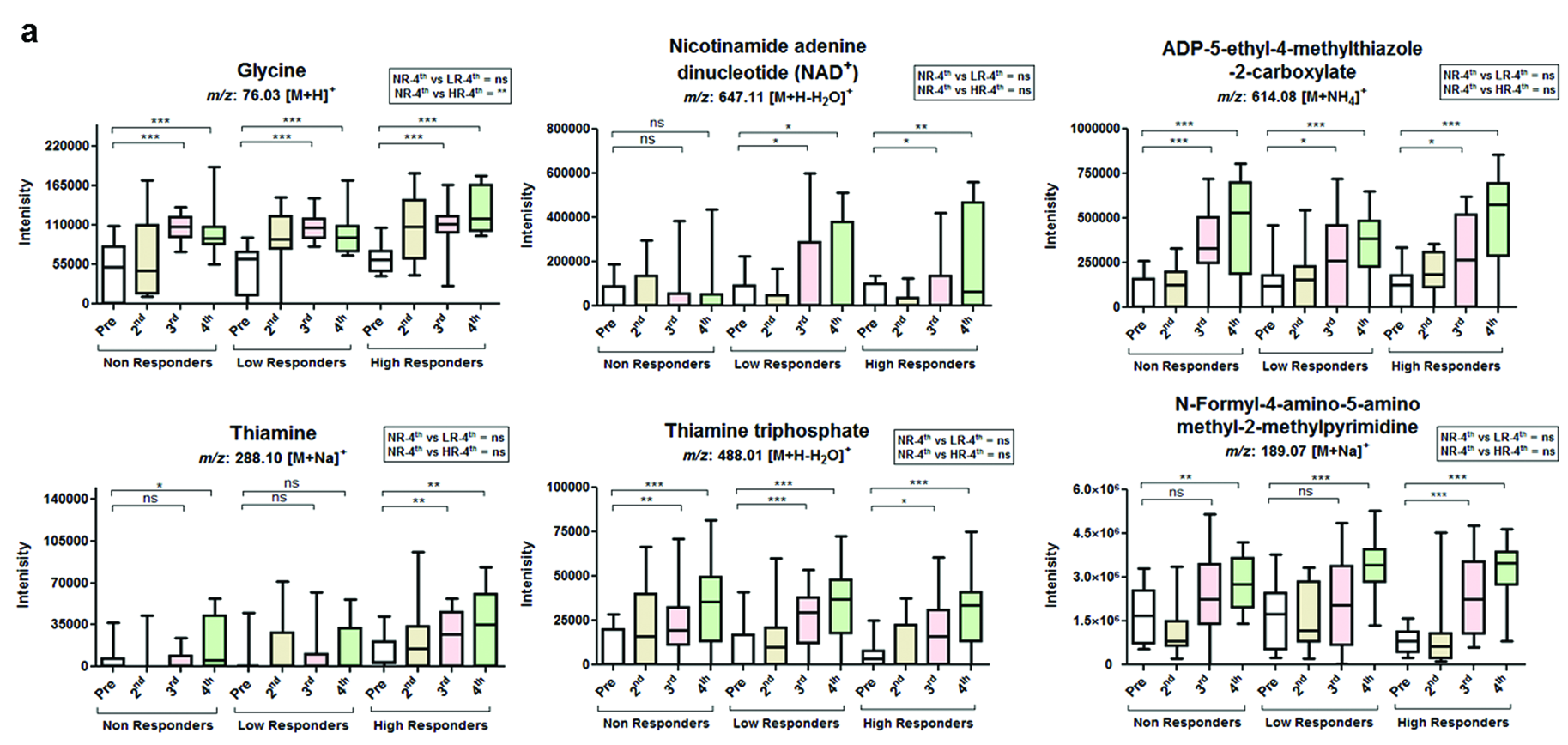
Supplementary fig S12.**

**Separately analyzed thiamine metabolism** **and pyrimidine metabolism pathway’s metabolites in non, low and high responders.** (a) Relative concentrations of significant upregulated metabolites in thiamine metabolism pathway detected in pre, 2^nd^, 3^rd^, and 4^th^, analyzed separately among NRs, LRs, and HRs vaccinees. (b) Relative concentrations of significant upregulated metabolites in pyrimidine metabolism detected in pre, 2^nd^, 3^rd^, and 4^th^, analyzed separately among NRs or LRs or HRs vaccinees. (c) Relative concentrations of significant upregulated metabolites in arachidonic acid metabolism pathway detected in pre, 2^nd^, 3^rd^, and 4^th^ vaccinees, analyzed separately among NRs, LRs, and HRs vaccinees. ****p* ≤ 0.001; ***p* ≤ 0.01; **p* ≤ 0.05; ^ns^not significant (*p* > 0.05)-student’s t-test, ^NR-4th^ non responders-4^th^ vaccinated,^L-4th^ low responders-4^th^ vaccinated , ^HR-4th^ high responders-4^th^ vaccinated.

**
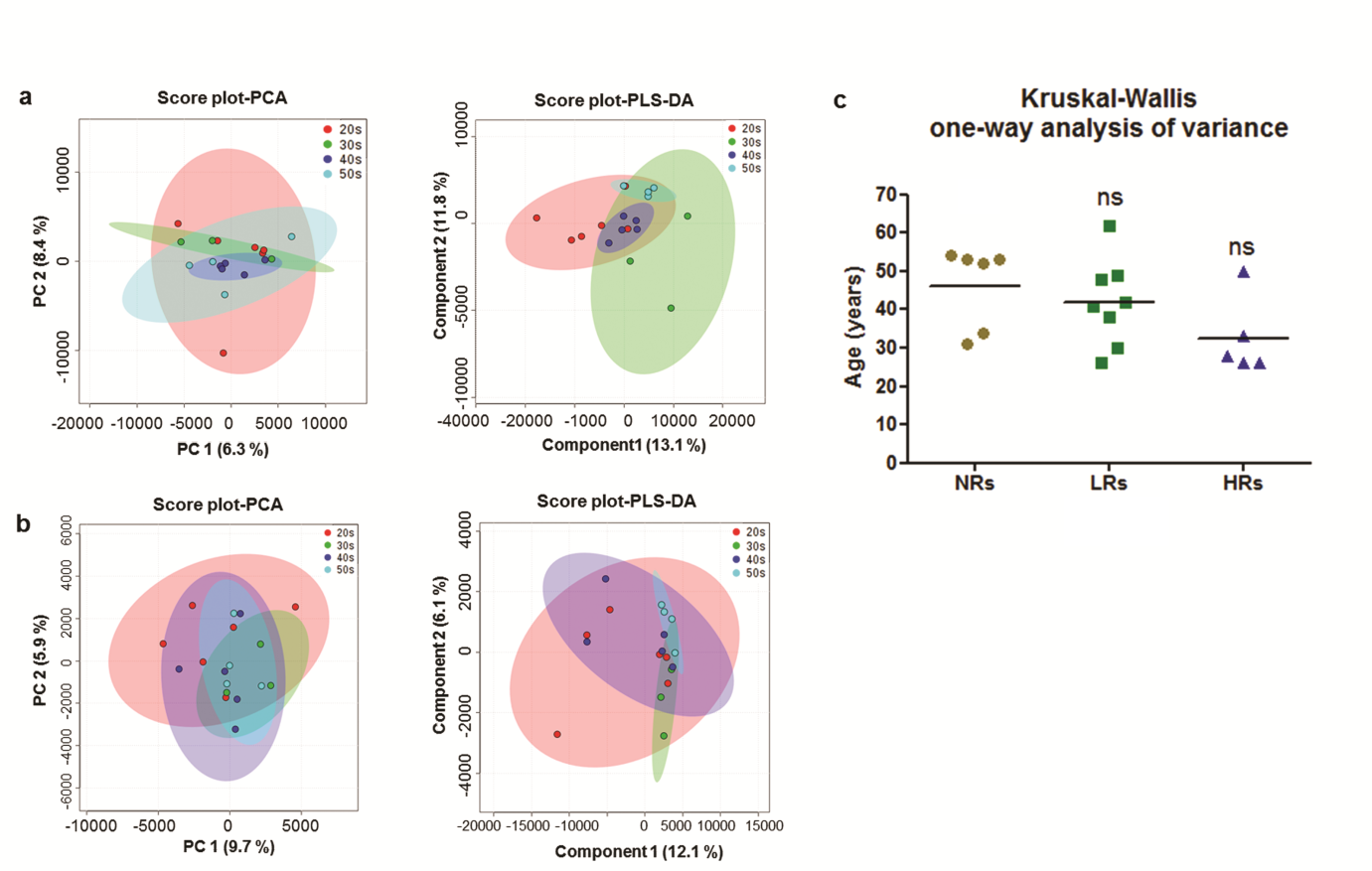
Supplementary fig S13.**

**
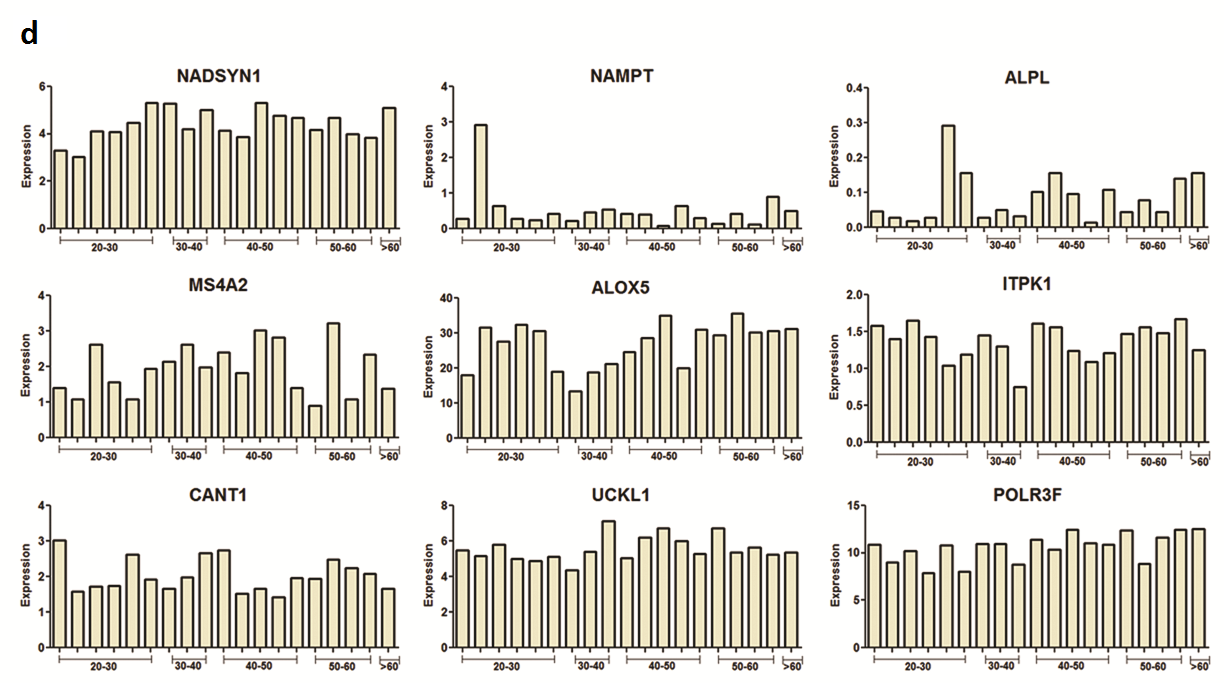
**

**Genes and metabolites analysis in age distributed vaccinees.** (a) PCA (left) and PLS-DA (right) analysis of raw gene expression among age distributed vaccinees using MetaboAnalyst 4.0. (b) PCA (left) and PLS-DA (right) analysis of raw (averaged) apLCMS features among age distributed vaccinees. In PCA and PLS-DA score plots, each data point represents individual vaccinee (c) Kruskal–Wallis one-way analysis of variance analysis in the age of NRs, LRs and HRs. (d) The raw expression of *NADSYN1*, *NAMPT*, *ALPL*, *MS4A2*, *ALOX5*, *ITPK1*, *CANT1*, *UCKL1*, *POLR3F* in age distributed vaccinees after 4^th^ vaccination. Each bar represents the individual vaccinees. No significant difference between age groups.

**
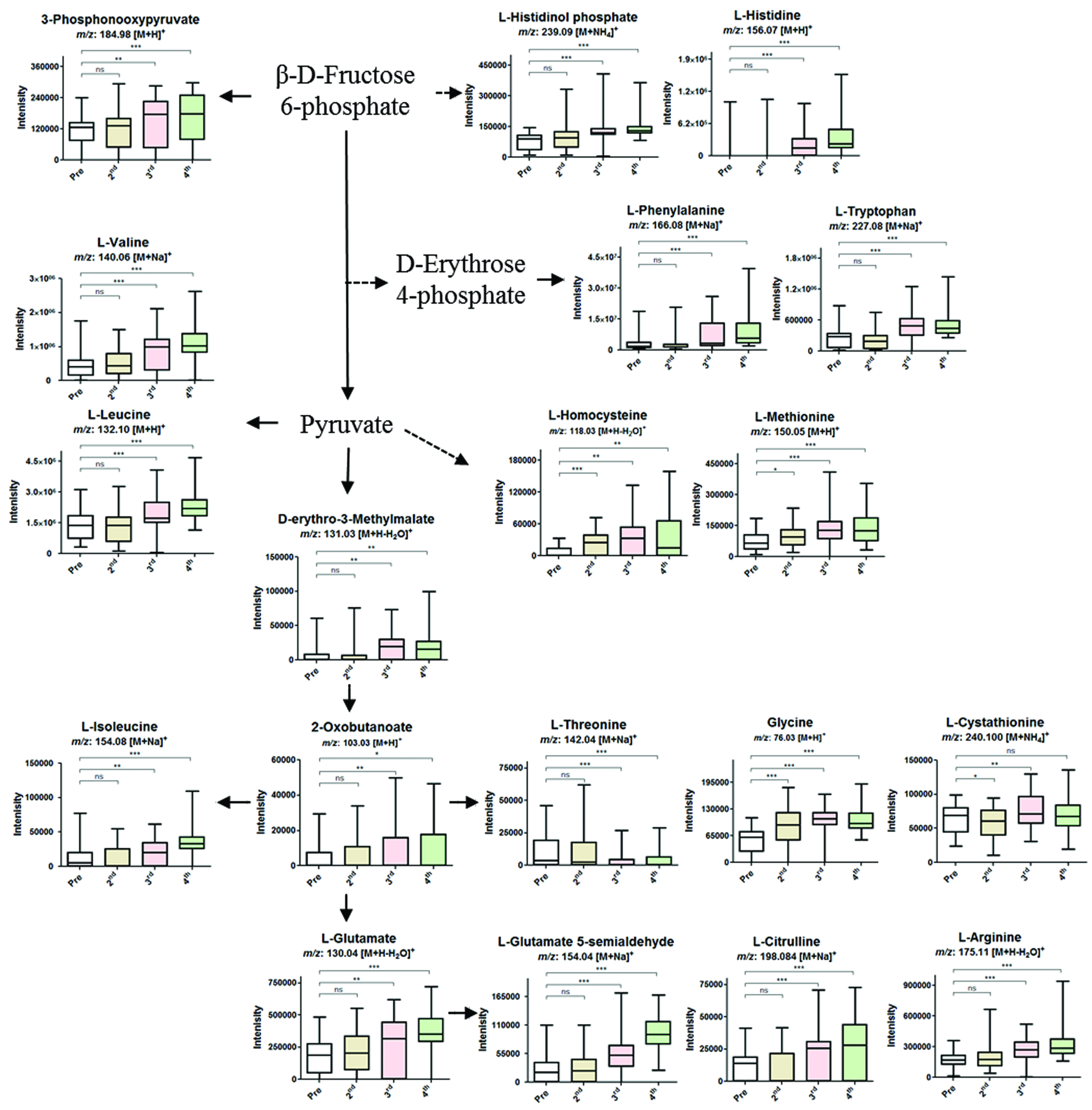
**

**Supplementary fig S14.**

**Pathway overview and relative concentrations of significant compounds in amino acid biosynthesis pathway**. Relative concentrations of significant upregulated 20 amino acids in amino acid biosynthesis pathway detected in pre, 2^nd^, 3^rd^, and 4^th^ vaccinees. ***p ≤ 0.001; **p ≤ 0.01; *p ≤ 0.05; ^ns^ represents non-significant (p > 0.05)-student’s t-test.
